# Supplementary material for: Mps1 Phosphorylates Its N-Terminal Extension to Relieve Autoinhibition and Activate the Spindle Assembly Checkpoint
Source: Curr Biol. 2018 Mar 19;28(6):872–883.e5. doi: 10.1016/j.cub.2018.02.002 (PMC5863767; doi:10.1016/j.cub.2018.02.002)
Supplement: Document S1. Figures S1–S7 [file mmc1.pdf]

Current Biology, Volume *28*

Supplemental Information

Mps1 Phosphorylates Its N-Terminal Extension to Relieve Autoinhibition and Activate the Spindle Assembly Checkpoint

Guillaume Combes, Helena Barysz, Chantal Garand, Luciano Gama Braga, Ibrahim Alharbi, Philippe Thebault, Luc Murakami, Dominic P. Bryne, Stasa Stankovic, Patrick A. Eyers, Victor M. Bolanos-Garcia, William C. Earnshaw, John Maciejowski, Prasad V. Jallepalli, and Sabine Elowe

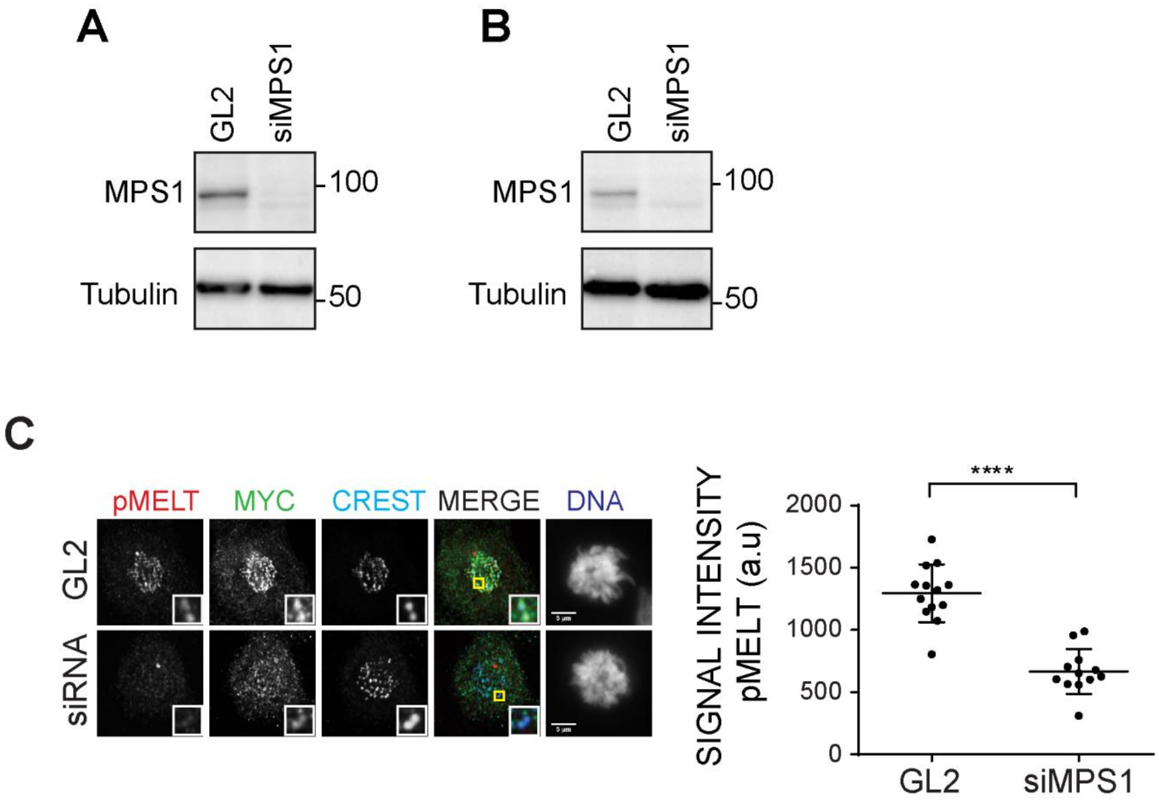

Figure S1: Verification of Mps1 siRNA efficiency, related to Figure 1.

siRNA GL2 (75nM) or siRNA Mps1-A (25nM)+Mps1-B (50nM) were transfected in either HeLa S3 **(A)** or HeLa T-Rex **(B)** cell lines for 72h before cells were lysed and cleared lysates were blotted for Mps1 (upper panels) and tubulin (lower panels). **(C)** pMELT signals in HeLa T-Rex cells depleted of endogenous Mps1. Cells were synchronized in mitosis after release from thymidine arrest before being fixed and immunostained for pMELT (Red) for, MYC1 (Green), CREST (Cyan) and Hoechst (Blue). Scale bar = 5µm. The graph shows the quantification of the relative pMELT intensity from. **** $P < 0.0001$.

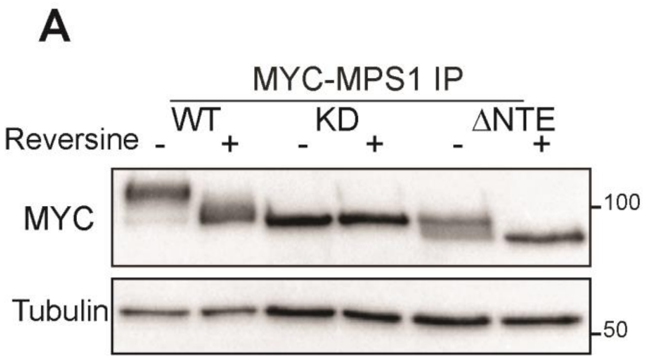

Figure S2: The NTE contributes to Mps1 mitotic hyperphosphorylation, related to Figure 1

(A) HEK 293T cells were transiently transfected with MYC-Mps1 constructs together with siMps1, and enriched in mitosis by nocodazole treatment. Cells were treated as indicated with 0.5 µM reversine 30min before harvesting. MYC (top panel) and α-tubulin (bottom panel) immunoblotting were performed on equalized lysates.

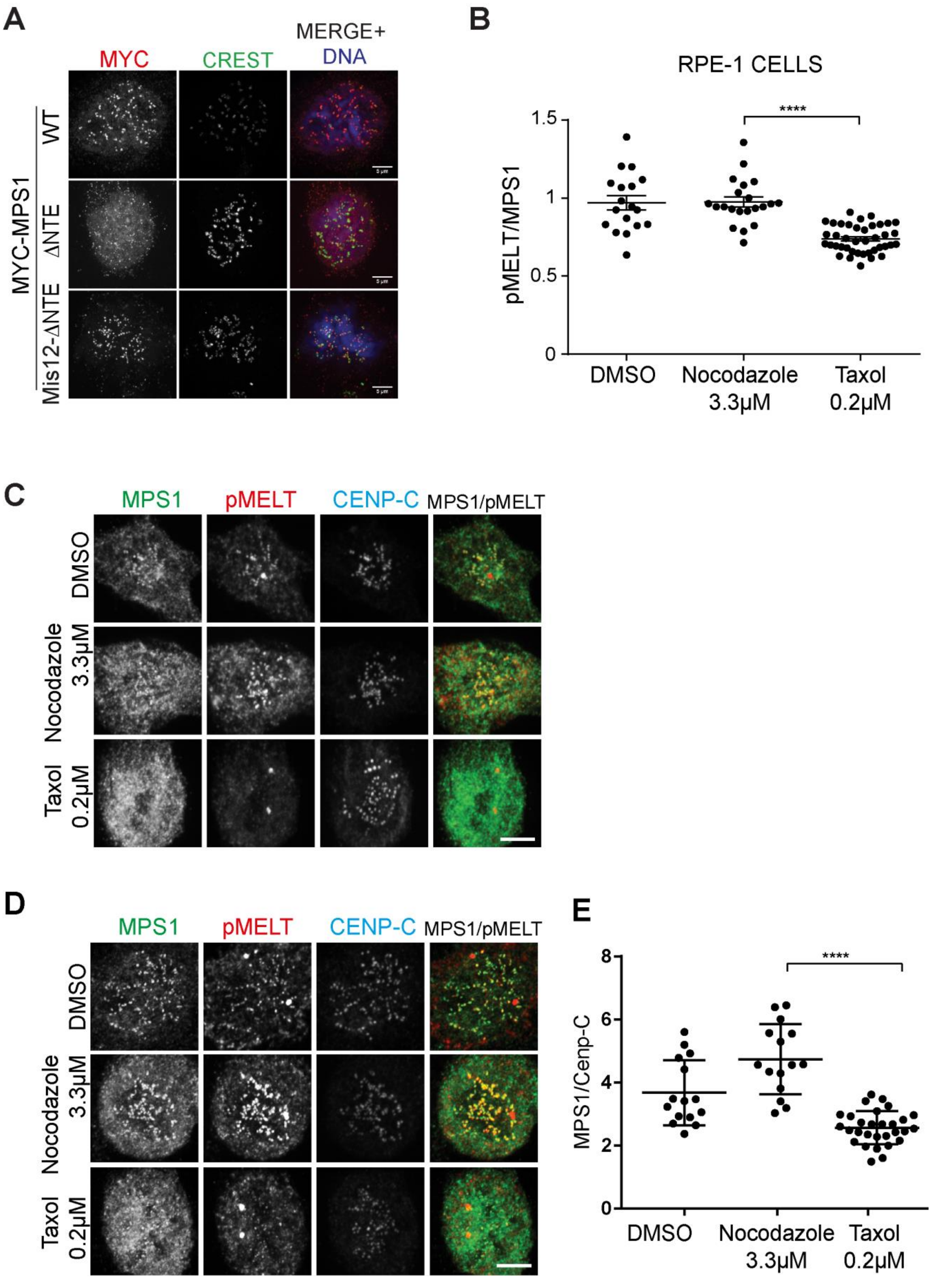

A
MYC
CREST
MERGE+
DNA
MYC-MPS1
WT
ΔNTE
Mis12-ΔNTE
5 µm
B
RPE-1 CELLS
pMELT/MPS1
1.5
1
0.5
0
****
DMSO
Nocodazole
3.3µM
Taxol
0.2µM
C
MPS1
pMELT
CENP-C
MPS1/pMELT
DMSO
Nocodazole
3.3µM
Taxol
0.2µM
D
MPS1
pMELT
CENP-C
MPS1/pMELT
DMSO
Nocodazole
3.3µM
Taxol
0.2µM
E
MPS1/Cenp-C
8
6
4
2
0
****
DMSO
Nocodazole
3.3µM
Taxol
0.2µM

Figure S3: Increased local concentration and transactivation contribute to Mps1 activation, related to Figure 2.

(A) HeLaS3 cells were simultaneously transfected with the indicated constructs and siMps1 and fixed 10h after release from a thymidine arrest. Cells were immunostained against MYC (Red), CREST (Green), and DNA was visualized by Hoechst (Blue). Scale bar = 5µm. **(B)** RPE-1 cells treated for 16h with 3.3µM nocodazole, 0.2µM taxol or DMSO, and then fixed. The graph shows the quantification of the pMELT signal relative to total Mps1. **** $p < 0.0001$. **(C)** Representative images of cells quantified in (B). Cells were immunostained against MYC (Green), pMELT (Red), CENP-C (Cyan) and Hoechst (Blue). Scale bar = 5µm. **(D)** HeLa cells treated for 16h with 3.3µM nocodazole, 0.2µM taxol or DMSO, and then fixed. Cells were immunostained against MYC (Green), pMELT (Red), CENP-C (Cyan) and DNA was marked by Hoechst (Blue). Scale bar = 5µm. See Figure 2F for quantification. **(E)** quantitation of the Mps1 signal versus Cenp-C at kinetochores for the data in Figure 2F.

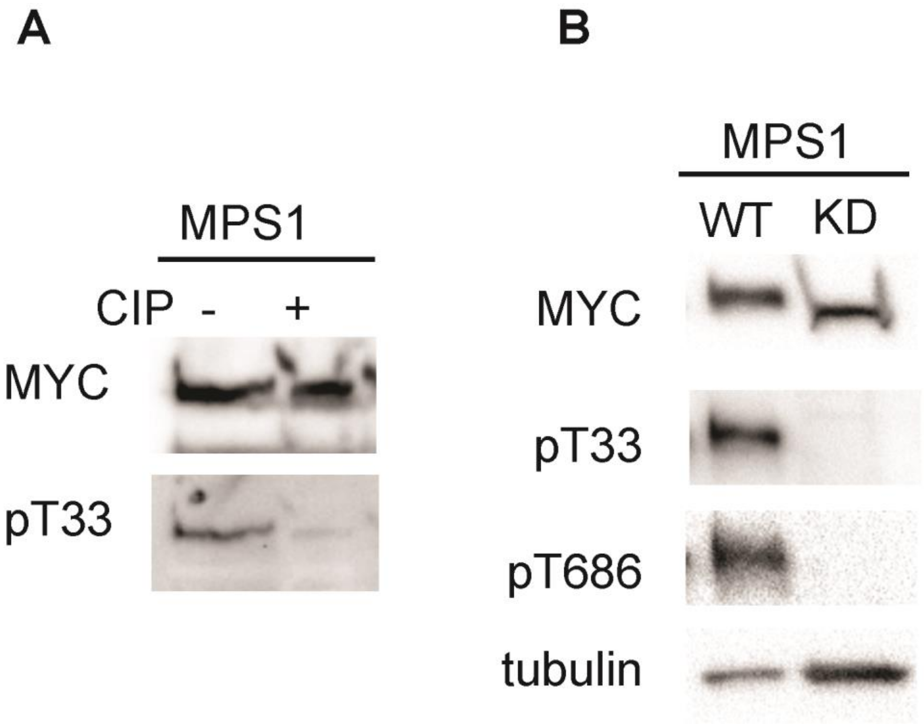

Figure S4. Characterization of the anti-pT33 phosphospecific Mps1 antibody, related to Figure 4.

(A) MYC-Mps1 was immunoprecipitated from nocodazole arrested HEK 293T cells and left untreated or was treated with calf-intestinal phosphatase for 60min at 37°C before the reaction was stopped with SDS-PAGE sample buffer. Resolved samples were probed with pT33 antibodies before stripping and reprobing with MYC. **(B)** Cells expressing MYC-Mps1 -WT and -KD were arrested in mitosis with nocodazole and the MYC-Mps1 proteins were immunoprecipitated from equalized lysates before being resolved by SDS-PAGE and immunoblotted with the indicated antibodies.

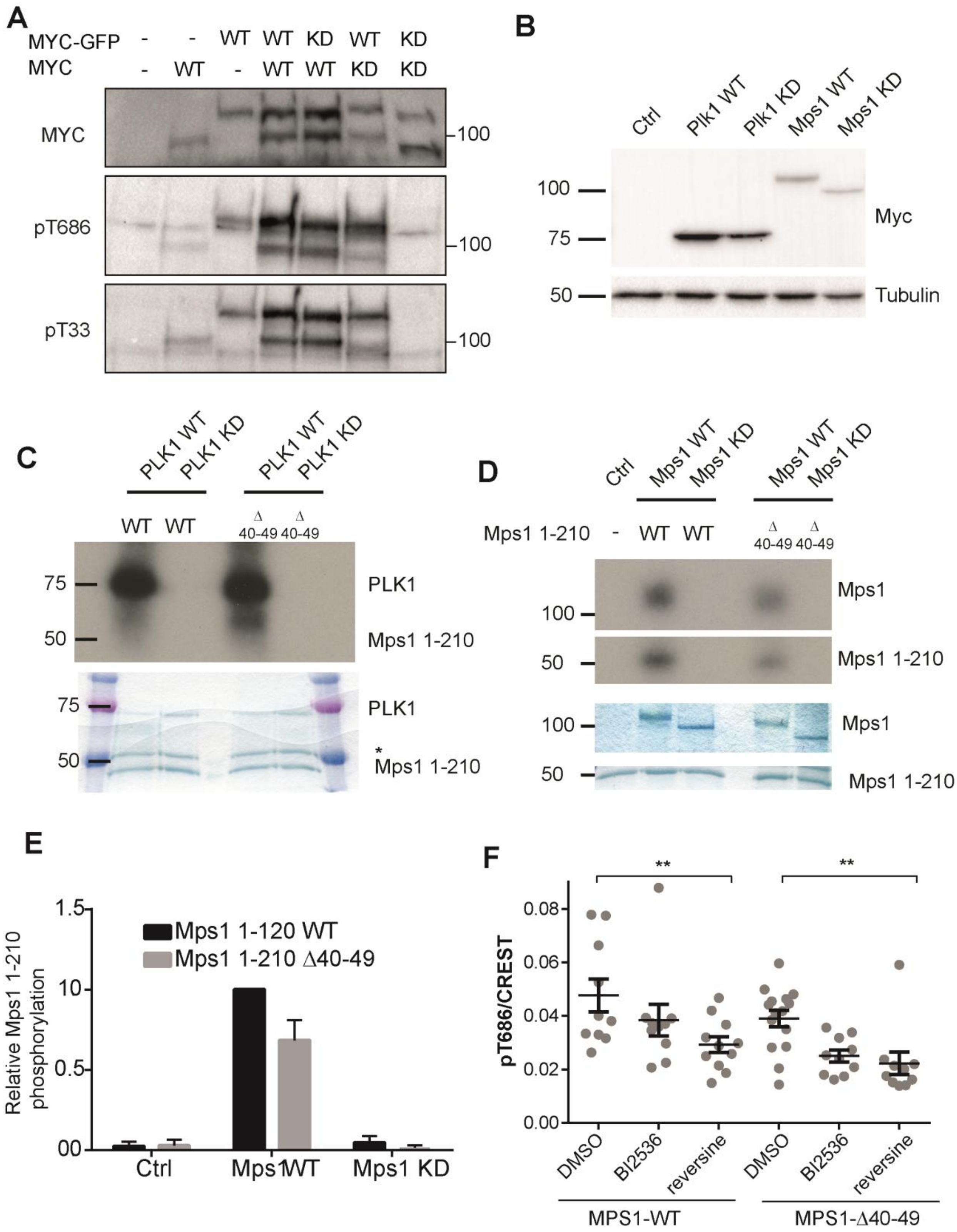

A
MYC-GFP - - WT WT KD WT KD
MYC - WT - WT WT KD KD
MYC
pT686
pT33
100
B
Ctrl
Plk1 WT
Plk1 KD
Mps1 WT
Mps1 KD
100
75
50
Myc
Tubulin
C
PLK1 WT
PLK1 KD
WT WT
Δ 40-49
75
50
PLK1
Mps1 1-210
D
Ctrl
Mps1 WT
Mps1 KD
Mps1 1-210 - WT WT
Δ 40-49
Mps1
Mps1 1-210
100
50
E
Mps1 1-120 WT
Mps1 1-210 Δ40-49
Relative Mps1 1-210 phosphorylation
1.5
1.0
0.5
0.0
Ctrl
Mps1 WT
Mps1 KD
F
pT686/CREST
0.08
0.06
0.04
0.02
0.00
**
DMSO
BI2536
reversine
MPS1-WT
MPS1-Δ40-49

Figure S5. Relief of Mps1 NTE-mediated autoinhibition does not require significant Plk1 activity, related to Figure 4.

(A) HEK 293T cells were transfected with the indicated Mps1-WT and -KD constructs. Lysates were blotted for T33 and T686 phosphorylation. Reblotting with MYC demonstrates equal loading. **(B)** Expression of Mps1 and Plk1 used in (C) and (D). In vitro kinase assay of Mps1 1-210 and Mps1 1-210 Δ40-49 recombinant fragments using full length Plk1 **(C)** and full length Mps1 **(D)**. **(E)** Quantitation of recombinant Mps1 fragment phosphorylation from (D). **(F)** MYC-GFP-Mps1 WT and Δ40-49 expressing HeLa cells were treated with nocodazole overnight before addition of MG132 and the additional inhibitors as indicated. Fixed cells were stained with MYC, Mps1 pT686, and CREST antisera. Relative quantification of pT686 signals is shown. **$p < 0.01$.

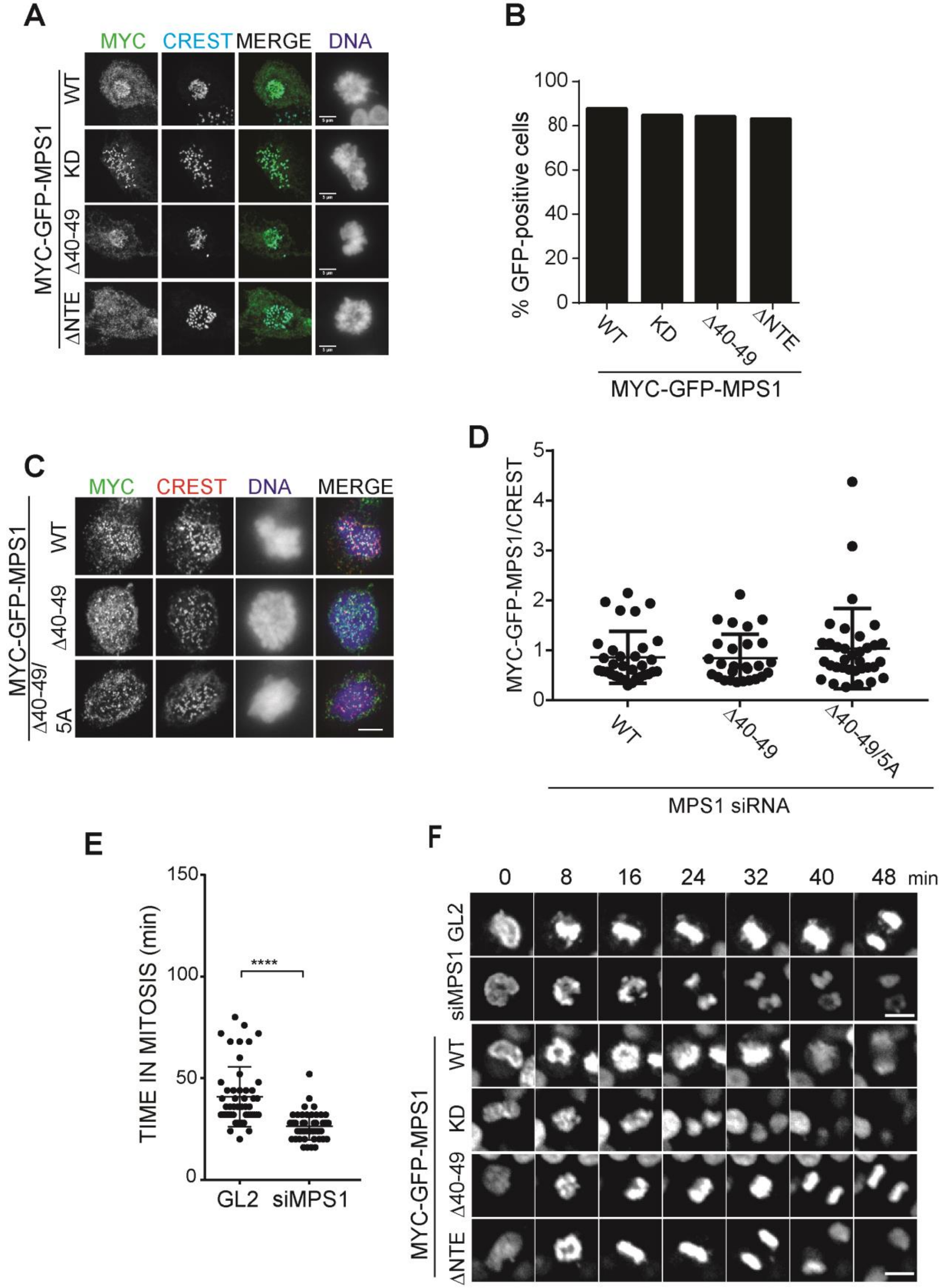

A
MYC
CREST
MERGE
DNA
MYC-GFP-MPS1
WT
KD
Δ40-49
ΔNTE
B
% GFP-positive cells
100
80
60
40
20
0
WT
KD
Δ40-49
ΔNTE
MYC-GFP-MPS1
C
MYC
CREST
DNA
MERGE
MYC-GFP-MPS1
WT
Δ40-49
Δ40-49/5A
D
MYC-GFP-MPS1/CREST
5
4
3
2
1
0
WT
Δ40-49
Δ40-49/5A
MPS1 siRNA
E
TIME IN MITOSIS (min)
150
100
50
0
****
GL2
siMPS1
F
0
8
16
24
32
40
48
min
GL2
siMPS1
MYC-GFP-MPS1
WT
KD
Δ40-49
ΔNTE

Figure S6. Characterization of the HeLa T-Rex stable cell lines used in this study, related to Figure 6.

(A) Immunofluorescence of HeLa T-Rex cell lines expressing 3xMYC-GFP-MPS1 constructs. Cells were stained for MYC (green), CREST (cyan) and DNA was visualized with Hoechst (blue). Scale bar=5µM. **(B)** Graphic representing the percentage of GFP-positive cells of the indicated cell lines. A minimum of 100 cells was counted per cell lines. **(C)** HeLa-T-Rex cell lines stably expressing the indicated constructs were enriched in mitosis by nocodazole treatment before fixation and staining with the indicated antibodies. **(D)** Quantitation of the relative kinetochore intensity of the Mps1 from the HeLa-T-Rex cell lines in (C). **(E)** Time (mins) in mitosis (nuclear envelope breakdown to anaphase onset) of siGL2 and siMPS1 treated cells, the control of the experiment in (Figure 6E). A minimum of 50 expressing cells was counted for each condition. n=2. Scale bar = 10µm. **** $p < 0.0001$. **(F)** Stills from representative cells of each cell line from live-cell experiments in (Figure 6E and Figure S6E).

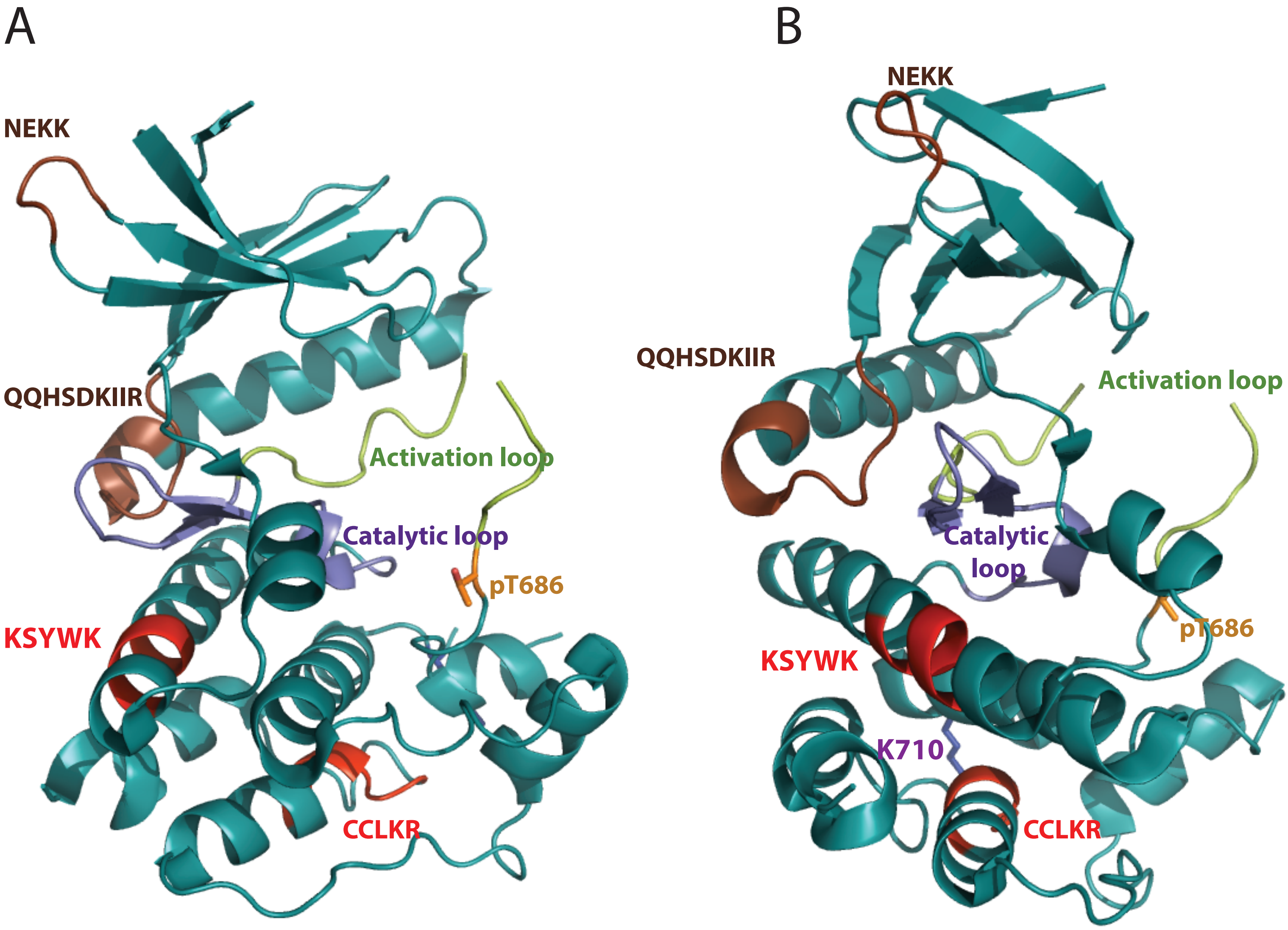
A
NEKK
QQHSDKIIR
Activation loop
Catalytic loop
pT686
KSYWK
CCLKR
B
NEKK
QQHSDKIIR
Activation loop
Catalytic loop
pT686
KSYWK
K710
CCLKR

Figure S7. Mps1 NTE and TPR crosslinks mapped onto the Mps1 kinase domain, related to Figure 7.

Unique crosslinks identified between the NTE and the kinase domain of Mps1 (brown) and the TPR and the kinase domain of Mps1 (red) are mapped on to the structure of the inhibited Mps1 kinase domain (PDB ID: 2ZMC). The catalytic loop is shown in blue whereas the activation loop is in green. **(A)** and **(B)** are rotated by 45º. Mapping the kinase domain residues identified in the cross-links with the NTE (and TPR) onto the Mps1 kinase structure suggests that the NTE region interacts with the N-terminal lobe region that is defined by the residues V^{539}FQVLNEKK^{547} and Q^{579}QHSDKIIR^{587} (numbering according to Mps1 isoform 1, NCBI NP_003309.2). The crystal structure of Mps1 kinase domain in its inhibited conformation shows that residues of the Q^{579}QHSDKIIR^{587} region form part of α-helix C and are in close spatial proximity (eg, 5 Å or less) to the activation and the catalytic loops. The C-terminal lobe region that is defined by the residues K^{625}SYWK^{629} and the more C-terminal region C^{770}CLKR^{774} both interact with the TPR motif. The activation loop (residues 672-680) and the loop between helices αF and αEF (residues 700-708) are flexible and disordered. The residues K^{625}SYWK^{629} form part of αE helix and are in close proximity to the catalytic loop (particularly the side chain of Tyrosine residue Y627). Because the C^{770}CLKR^{774} residues form part of αH helix, the intramolecular interaction of the TPR motif with the kinase domain may affect the local conformation of the activation loop, thus contributing to modulate Mps1 catalytic activity. Two positively charged residues, K708 and K710, which are mapped onto the loop connecting αF and αEF and in close proximity to the P+1 and the activation loops, stabilize the active conformation.
