# Supplementary material for: Mps1 Phosphorylates Its N-Terminal Extension to Relieve Autoinhibition and Activate the Spindle Assembly Checkpoint
Source: Curr Biol. 2018 Mar 19;28(6):872–883.e5. doi: 10.1016/j.cub.2018.02.002 (PMC5863767; doi:10.1016/j.cub.2018.02.002)
Supplement: Document S2. Article plus Supplemental Information [file mmc8.pdf]

Article

Current Biology

Mps1 Phosphorylates Its N-Terminal Extension to Relieve Autoinhibition and Activate the Spindle Assembly Checkpoint

Highlights

- Mps1 autophosphorylation at the NTE promotes activity independent of localization
- NTE phosphorylation relieves an NTE-dependent autoinhibition
- Mps1 autophosphorylation at its NTE is essential for the SAC, but not congression

Authors

Guillaume Combes, Helena Barysz, Chantal Garand, ..., John Maciejowski, Prasad V. Jallepalli, Sabine Elowe

Correspondence

sabine.elowe@crchuq.ulaval.ca

In Brief

Combes et al. demonstrate that autophosphorylation by the spindle checkpoint kinase Mps1 at its N-terminal extension relieves catalytic autoinhibition and is required for optimal checkpoint activity. This occurs independently of the role of the N-terminal extension in kinetochore binding, suggesting multiple roles for this region during mitosis.

Check for updates

Combes et al., 2018, Current Biology *28*, 872–883
March 19, 2018 © 2018 The Author(s). Published by Elsevier Ltd.
https://doi.org/10.1016/j.cub.2018.02.002

CellPress

CellPress

Current Biology
Article

Mps1 Phosphorylates Its N-Terminal Extension to Relieve Autoinhibition and Activate the Spindle Assembly Checkpoint

Guillaume Combes,[1,2] Helena Barysz,[3,7] Chantal Garand,[2] Luciano Gama Braga,[1,2] Ibrahim Alharbi,[1,2] Philippe Thebault,[2] Luc Murakami,[2] Dominic P. Bryne,[4] Stasa Stankovic,[5] Patrick A. Eyers,[4] Victor M. Bolanos-Garcia,[5] William C. Earnshaw,[3] John Maciejowski,[6] Prasad V. Jallepalli,[6] and Sabine Elowe[1,2,8,*]

[1]Programme in Molecular and Cellular Biology, Faculty of Medicine, Université Laval, 1050 Avenue de la Médecine, Bureau 4633, Université Laval, Québec, QC G1V0A6, Canada

[2]Axe of Reproduction, Mother and Youth Health, Centre de Recherche du Centre Hospitalier Universitaire de Québec, Québec, QC G1V 4G2, Canada

[3]Wellcome Centre for Cell Biology, Institute of Cell Biology, University of Edinburgh, Edinburgh EH9 3BF, UK

[4]Department of Biochemistry, Institute of Integrative Biology, University of Liverpool, Liverpool L69 7ZB, UK

[5]Department of Biological and Medical Sciences - Faculty of Health and Life Sciences, Oxford Brookes University, Oxford OX3 0BP, UK

[6]Molecular Biology Program, Sloan Kettering Institute, Memorial Sloan Kettering Cancer Center, New York, NY 10065, USA

[7]Present address: Lonza Group AG, 4052 Basel, Switzerland

[8]Lead Contact

*Correspondence: sabine.elowe@crchuq.ulaval.ca

https://doi.org/10.1016/j.cub.2018.02.002

SUMMARY

Monopolar spindle 1 (Mps1) is a conserved apical kinase in the spindle assembly checkpoint (SAC) that ensures accurate segregation of chromosomes during mitosis. Mps1 undergoes extensive auto- and trans-phosphorylation, but the regulatory and functional consequences of these modifications remain unclear. Recent findings highlight the importance of intermolecular interactions between the N-terminal extension (NTE) of Mps1 and the Hec1 subunit of the NDC80 complex, which control Mps1 localization at kinetochores and activation of the SAC. Whether the NTE regulates other mitotic functions of Mps1 remains unknown. Here, we report that phosphorylation within the NTE contributes to Mps1 activation through relief of catalytic autoinhibition that is mediated by the NTE itself. Moreover, we find that this regulatory NTE function is independent of its role in Mps1 kinetochore recruitment. We demonstrate that the NTE autoinhibitory mechanism impinges most strongly on Mps1-dependent SAC functions and propose that Mps1 activation likely occurs sequentially through dimerization of a "prone-to-autophosphorylate" Mps1 conformer followed by autophosphorylation of the NTE prior to maximal kinase activation segment trans-autophosphorylation. Our observations underline the importance of autoregulated Mps1 activity in generation and maintenance of a robust SAC in human cells.

INTRODUCTION

Cell division is orchestrated by a precise and highly regulated series of events. To avoid errors in chromosome segregation during mitosis and meiosis, the cell has evolved a signaling mechanism called the spindle assembly checkpoint (SAC) [1–3]. The SAC monitors the robustness of spindle microtubule attachments during mitosis and delays the onset of anaphase until all sister chromatids become appropriately attached. SAC activity initiates at kinetochores, complex protein structures that form at centromeres and mediate the interaction between dividing sister chromatids and spindle microtubules during mitosis [2, 3]. Microtubule capture is thought to occur at the outer kinetochore by the KMN network (KNL1 complex, NDC80 complex, and MIS12 complex) [4], and some components of this network, in particular Hec1 and Knl1, are directly involved in SAC establishment. Knl1 is a major signaling hub during mitosis, and its phosphorylation by Mps1, or alternatively by Plk1, under conditions where Mps1 activity is compromised or does not exist as in certain nematodes, initiates SAC signaling. In contrast, dephosphorylation by a PP2A-PP1 relay is thought to be a major mechanism of attenuating the SAC [5–7].

Mps1 has emerged as a master conductor of SAC signaling and is one of the first protein kinases recruited to unattached kinetochores in early mitosis [8]. Mps1 activity is regulated by a cooperative series of auto- and transphosphorylation reactions that appear to be dependent on Mps1 and Plk1 [6, 9–11]. Activated Mps1 phosphorylates the KNL complex at conserved Thr residues in so-called MELT (for Met-Glu-Leu-Thr core consensus) repeat motifs. This generates high-affinity binding sites for Bub3 and promotes recruitment of a Bub1-Bub3-BubR1-Bub3 heterotetrameric complex to kinetochores [12–19]. Recent reports suggest that phosphorylation of Bub1 by Mps1 promotes its binding to the Mad1-Mad2 heterodimer [20–22]. Mad1-Mad2 complex formation and activation occurs through a templating mechanism and involves the conversion of an "open" O-Mad2 to a SAC-competent, "closed" C-Mad2 conformation [23]. Mps1 activity is required for retaining both the Mad1-C-Mad2 complex and O-Mad2 at

This is an open access article under the CC BY license (http://creativecommons.org/licenses/by/4.0/).

unattached kinetochores during mitosis [24]. In line with these pleotropic functions, depletion of Mps1 [25, 26] or chemical inhibition of its catalytic activity [9, 27, 28] confirmed a requirement for Mps1 in proper chromosome alignment and accurate chromosome segregation.

Critical to human Mps1 activity is its localization to the kinetochore, which in human cells is primarily mediated by the non-catalytic NTE (N-terminal extension), with a minor contribution from the MR (middle region). Together, these regions sandwich a TPR (tetratricopeptide repeat) motif, which also plays a role in Mps1 localization [29–34]. The NTE is a region of about fifty amino acids at the extreme N terminus; it is rather poorly conserved and lacks clear structural and functional domains. Recent evidence suggests that human Mps1 localization is primarily controlled by the NTE through competition with microtubules for their binding site on the calponin homology (CH) domain of Hec1 [35], although budding yeast Mps1 can also directly associate with attached kinetochores [36]. The Mps1 MR contributes to Mps1 localization through interactions with the CH domain of Nuf2 that appear to be regulated by Aurora B phosphorylation [32, 35, 37]. Despite poor conservation, the N terminus of fission yeast Mph1 is also required for its kinetochore recruitment to the NDC80 complex, albeit by potentially different mechanisms [38, 39]. Curiously, in budding yeast, the NTE has been reported to contribute to Mps1 kinase activity [40]. Our previous studies of human Mps1 also confirm that the N terminus of Mps1 promotes catalytic activation [30], although whether this functionality is associated with the NTE or the TPR remains unclear [29–31]. Taken together, these observations raise the intriguing possibility that the NTE of human Mps1 may be involved in the regulation of Mps1 kinase activity, in addition to a well-established role in kinetochore docking.

In this study, we found that the NTE contributes to Mps1 catalytic activation that is independent of its role in kinetochore docking. We show that residues 40–49 in the NTE are required for full kinase activation independent of Hec1 binding. In particular, phosphorylation of this region promotes relief of NTE-specific autoinhibition, which is required for optimal SAC activity. An Mps1 mutant protein lacking residues 40–49 and mutated at adjacent phosphorylation sites fully restores kinase and SAC function, suggesting that multiple phosphorylation events in the Mps1 NTE are required for complete activation of Mps1. Together, our data confirm that the Mps1 NTE contributes to robust activation of the SAC through relief of autoinhibition, in addition to its established role in kinetochore binding.

RESULTS

Mps1-ΔNTE Exhibits Both Attenuated Activity and Kinetochore Localization

To evaluate the potential role of the NTE in Mps1 localization and catalytic activity, we generated an Mps1 construct lacking the first 49 amino acids (Mps1-ΔNTE; Figure 1A). In agreement with previous studies [31], MYC-Mps1-ΔNTE localized weakly at kinetochores after depletion of endogenous Mps1 (Figure S1), even after inhibition of Mps1 activity with reversine, which decreases Mps1 turnover at kinetochores [41] (Figure 1B). This extends previous reports and establishes that the effect of Mps1 activity on localization is negligible in the absence of the NTE. As a measure of Mps1 activation, we used phosphospecific antibodies recognizing Mps1 activation loop pT676 and pT686 [6, 11]. In cells depleted of endogenous Mps1, MYC-Mps1 wild-type (WT) exhibited robust phosphorylation at the activation loop (T676) and P+1 loop (T686) sites (together referred to as the activation segment), whereas both MYC-Mps1 kinase-dead (KD) (D664A) and MYC-Mps1-ΔNTE exhibited much lower levels of phosphorylation (Figure 1C). Phosphorylation of a proline-directed site (S821) was only marginally decreased in MYC-Mps1-ΔNTE. Mps1-ΔNTE also exhibited attenuated gel retardation when compared with Mps1-WT, characteristic of reduced activity-dependent autophosphorylation (Figure S2). Finally, we measured *in vitro* catalytic output using both Mps1 autophosphorylation and phosphorylation of MBP (myelin basic protein) (Figures 1D and 1E). As expected, Mps1-WT was efficient at both auto- and substrate phosphorylation, whereas Mps1-KD was completely ineffective at both. Surprisingly, and in contrast to observations with the phosphospecific antibodies in cell extracts, the isolated Mps1-ΔNTE phosphorylated both itself and MBP as efficiently as Mps1-WT.

Mps1 Clustering by the NTE Supports Mps1 Kinase Activity

One possible interpretation of the discordance between the results obtained with phosphospecific antibodies and *in vitro* kinase assays is that a high degree of antibody-mediated Mps1 clustering induced by immunoprecipitation prior to the *in vitro* kinase assay may serve to promote Mps1 autoactivation *in trans* [9, 10, 26, 42, 43]. A cellular function of Mps1 kinetochore localization might therefore be increasing the local concentration of Mps1 to trigger autoactivation [42]. To test this idea, we expressed Lac repressor (LACI) fusion proteins with Mps1 in U2OS cell lines expressing 256 copies of the Lac operator in chromosome 1 [44]. In nocodazole-arrested cells, MYC-LACI-Mps1-WT (but not Mps1-KD) was phosphorylated at T686, as expected. In contrast, MYC-ΔNTE was very poorly phosphorylated at this site, whereas MYC-LACI-Mps1-ΔNTE was able to autophosphorylate efficiently (Figure 2A). These results were confirmed using quantitative immunofluorescence (Figures 2B and 2C). In agreement, forced kinetochore localization of Mps1-ΔNTE via N-terminal fusion to the kinetochore component Mis12 (Figure S3A) restored Mps1-ΔNTE autophosphorylation at both T686 and T676 to levels comparable to Mps1-WT (Figure 2D). To test the idea that dimerization of Mps1-ΔNTE may promote its activity regardless of subcellular localization, we exploited chemical-induced dimerization. To accomplish this, we generated Mps1 proteins fused to the FK506-binding protein (FKBP), which homodimerizes in the presence of the small-molecule ligand, AP20187. In nocodazole-arrested cells treated with AP20187, MYC-Mps1-ΔNTE was poorly phosphorylated, whereas AP20187 treatment resulted in a rescue of autophosphorylation at T686 in MYC-FKBP-Mps1-ΔNTE-expressing cells to levels similar to those observed with Mps1-WT. As expected, MYC-FKBP-Mps1-KD was not autophosphorylated at T686 (Figure 2E). Thus, enforced Mps1-ΔNTE dimerization in the cytoplasm during mitosis is sufficient to induce its activation, confirming the importance of Mps1 clustering for activation through promotion of autophosphorylation.

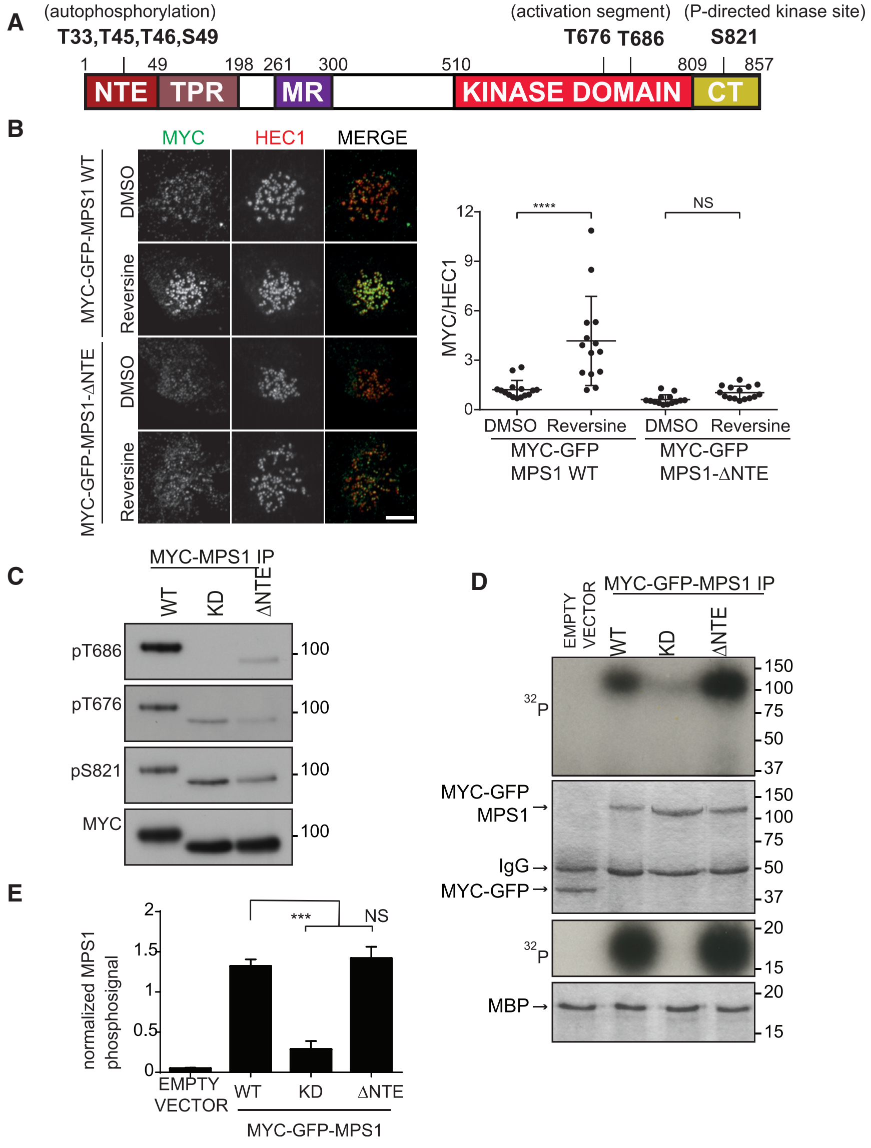

Figure 1. Mps1-ΔNTE Exhibits Attenuated Activity and Kinetochore Localization

(A) Mps1 domains and phosphorylation sites relevant to this study.

(B) Mitotic cells expressing Myc-GFP-Mps1 WT and ΔNTE and siMps1 were treated and immunostained as indicated. The scale bar represents 5 μm. Quantification shows the MYC-Mps1/Hec1 ratio. ****p < 0.0001.

(C) Phosphorylation of MYC-Mps1-WT, -KD, or -ΔNTE expressed in mitotic HEK293T cells together with Mps1 small interfering RNA (siRNA).

(D) *In vitro* kinase assay of MYC-GFP-Mps1-WT, -KD, and -ΔNTE expressed as in (C) and visualized by autoradiography (first and third panels). Coomassie Blue shows equal loading.

(E) Quantification of Mps1 autophosphorylation from (D).

Data are means ± SEM from n = 2 independent experiments. ***p < 0.001. See also Figures S1 and S2.

To provide functional evidence for this mechanism, we verified the effect of clustering endogenous Mps1 at kinetochores by measuring phosphorylation of the physiological Mps1 substrate Knl1 with pT875 phosphospecific antibodies (hereafter referred to as pMELT) [13, 14, 16–19]. Treatment with Taxol and nocodazole has been proposed to differentially affect Mps1 recruitment levels; Taxol-hyperstabilized microtubules are thought to compete with (and therefore decrease) Mps1 localization at kinetochores, whereas nocodazole destabilizes microtubule interactions associated with increased recruitment of Mps1 to kinetochores [35]. We confirmed these observations (Figure S3E) and demonstrated that, in both HeLa S3 (Figures 2F and S3D) and RPE-1 (Figures S3B and S3C) cells, pMELT:Mps1 signals at kinetochores were significantly lower in Taxol-arrested cells when compared to nocodazole-arrested cells. Collectively, these data imply that there is a negative correlation between Mps1 kinetochore occupancy and substrate phosphorylation and that NTE-mediated kinetochore localization contributes to Mps1 activation.

Dissection of the NTE Reveals an Autoinhibitory Motif Regulating Kinase Activity

Our data thus far suggest that the Mps1 NTE promotes high local concentrations of the kinase at kinetochores, which contributes to its own optimal activation. We next sought to determine the specific region of the NTE that drives this process. Although the NTE is rather poorly conserved, N-terminal alignment of multicellular eukaryote sequences revealed three short regions of conservation within the first 49 amino acids that we reasoned might be important for NTE functions (Figure 3A). We therefore generated a panel of different Mps1 mutants: the deletion mutants Mps1-Δ7, Mps1-Δ19–29, and Mps1-Δ40–49, as well as Mps1-5A, which consists of alanine mutations of a conserved acidic region (30-31-33-34-35), including the previously identified autophosphorylation site T33 [45].

Using phosphospecific Mps1 antibodies, we tested phosphorylation of these mutants together with Mps1-ΔTPR. As expected, Mps1-WT, but not Mps1-KD, was phosphorylated at both T686 and T676, whereas Mps1-ΔNTE displayed markedly decreased autophosphorylation as described above. Mps1-Δ7, Mps1-Δ19–29, and Mps1-5A displayed only very slightly reduced levels of Mps1 autophosphorylation compared to Mps1-WT. In contrast, Mps1-Δ40–49 and Mps1-ΔTPR exhibited decreased phosphorylation profiles that were similar to Mps1-ΔNTE (Figure 3B). Individual HeLa cells depleted of endogenous Mps1 and expressing Mps1-Δ40–49 also displayed a significantly attenuated pT686 signal compared to cells rescued with Mps1-WT (Figure 3C). This suggests that, whereas multiple regions of the NTE may incrementally contribute to Mps1 activity, residues 40–49 in the NTE play a more significant role in Mps1 catalytic autoactivation.

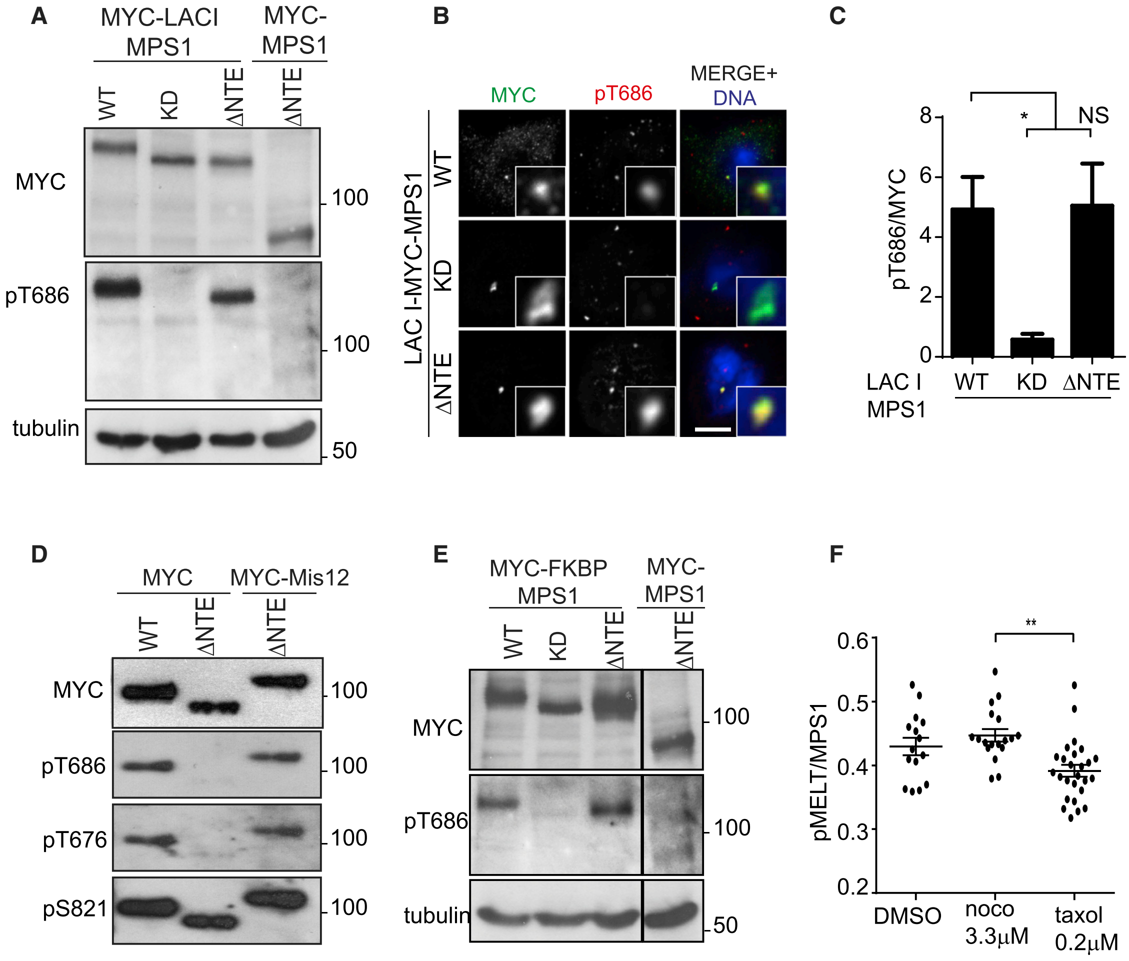

Figure 2. Mps1 Clustering in the NTE Supports Its Kinase Activity

(A) Immunoblotting with the indicated antibodies of MYC-LacI-Mps1-WT, -KD, -ΔNTE, or MYC-Mps1-ΔNTE expressed with siMps1 in a U2OS line expressing a LAC^{O} array and enriched in mitosis.

(B) Cells were treated as in (A) but fixed for immunofluorescence as indicated. The scale bar represents 5 μm.

(C) Quantification of pT686/MYC-Mps1 ratios from (B). $^{*}p < 0.05$; $n = 3$.

(D) Immunoblotting was performed with the antibodies shown on lysates from cells expressing the indicated Mps1 constructs.

(E) Cells were transfected with the indicated MYC-FKBP-Mps1 constructs together with siMps1, synchronized in mitosis, and treated with 10 nM AP20187 1 hr before immunoblotting as indicated. Note that an intermittent band has been excised out.

(F) HeLa cells were treated overnight with the indicated drugs before fixation. Quantification of pMELT/Mps1 ratios is shown. $^{**}p < 0.01$.

See also Figure S3.

Our data reveal that Mps1 clustering at the kinetochore via the NTE promotes its activation, and so we next sought to determine the subcellular localization of Mps1-Δ40–49, reasoning that these residues might contribute to Hec1 binding, which is required for optimal kinetochore association. We examined the localization of the Mps1 mutants while concomitantly depleting endogenous Mps1 to prevent dimerization with the endogenous protein. Surprisingly, Mps1-Δ40–49 behaved like Mps1-WT, whereas Mps1-ΔNTE localized very weakly to kinetochores in the absence or presence of reversine (Figures 3D and 3E), as previously established (Figure 1B). In agreement with these observations, Mps1-ΔNTE associated more weakly with recombinant GST-CH^{HEC1} in pull-down experiments, whereas Mps1-WT and Mps1-Δ40–49 were bound to GST-CH^{HEC1} at similar levels (Figure 3F). Collectively, our data suggest that, whereas Mps1 kinetochore docking helps to promote catalytic activity, the enzymatic and localization functions of the Mps1-NTE can be uncoupled.

Residues 40–49 Form an NTE-Specific Kinase Autoinhibitory Module

Considering that immunoprecipitation and clustering of Mps1-ΔNTE restored catalytic activity, we reasoned that NTE residues 40–49 might be involved in Mps1 dimerization and catalytic activation. Similar reasoning also led us to hypothesize that clustering by immunoprecipitation of the Mps1-ΔTPR deletion mutant, which localizes less efficiently than Mps1-WT to kinetochores [31], might restore activity. Consistently, Mps1-ΔNTE and Mps1-ΔTPR demonstrated levels of activity equivalent to Mps1-WT when immunoprecipitated and subjected to *in vitro*

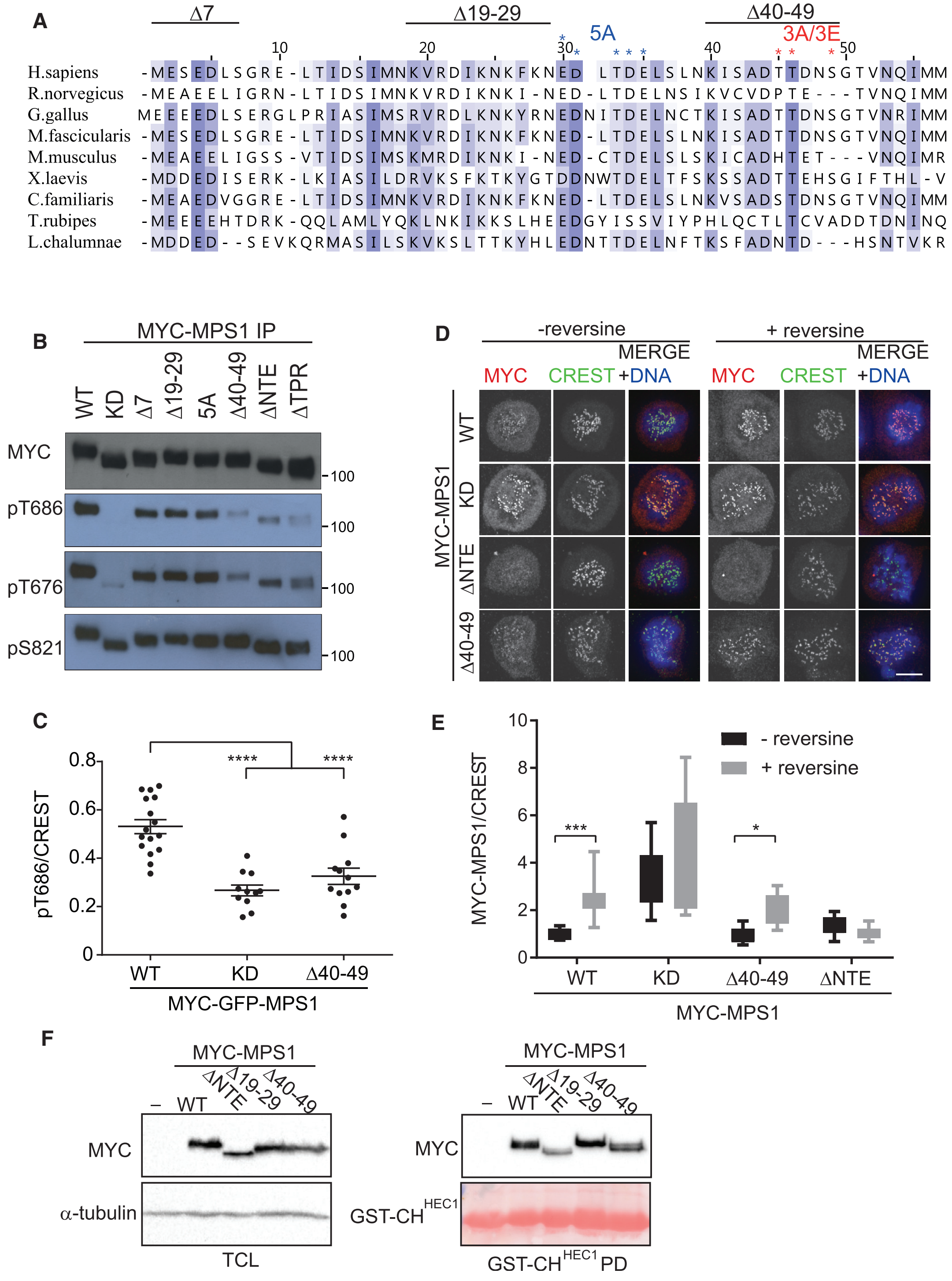

Figure 3. Dissection of the NTE Reveals an Autoinhibitory Motif Regulating Kinase Activity

(A) Evolutionary conservation of the Mps1 NTE and deletion mutants generated for this study. Residues targeted are indicated with an asterisk.

(B) HEK293T cells were transfected with the indicated plasmids together with siMps1 before enrichment in mitosis and immunoblotting with the indicated antibodies.

(C) Cells expressing indicated Mps1 constructs and siMps1 were synchronized in mitosis before fixation and immunostaining as shown. Quantification of pT686/CREST ratios is shown. ****$p < 0.0001$.

(D) HeLa cells expressing the indicated constructs, together with siMps1, were fixed and immunostained as indicated. The scale bar represents 5 μm.

(E) Quantification of the MYC-Mps1/CREST ratio from (D); *$p < 0.05$; ***$p < 0.001$.

(F) Pull-down of indicated MYC-GFP-tagged Mps1 with GST-CH^{HEC1}.

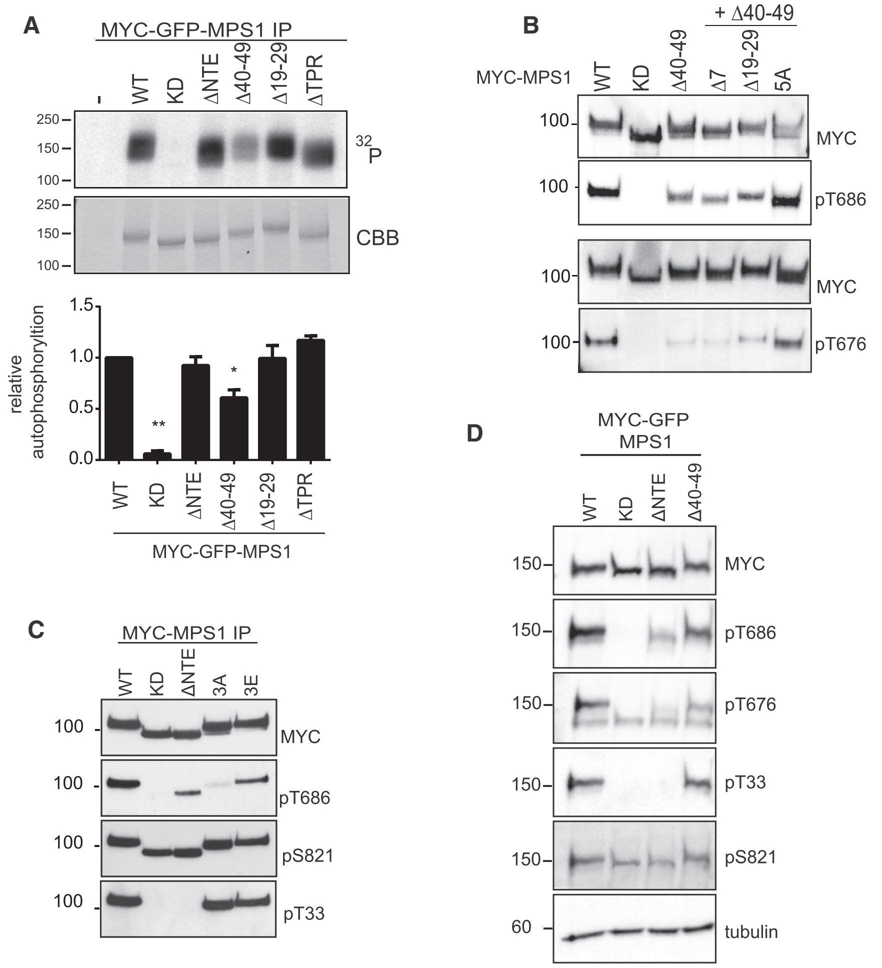

Figure 4. Residues 40–49 Form an NTE-Specific Kinase Autoinhibitory Module

(A) *In vitro* kinase assay of MYC-GFP-Mps1 constructs. Coomassie blue staining indicated equal loading. Quantification of Mps1 autophosphorylation is shown; data are means ± SEM; n = 3. *p < 0.05 and **p < 0.01.

(B) Immunoblotting with the indicated antibodies of the various MYC-Mps1 mutant proteins.

(C) MYC immunoprecipitates from cells expressing the indicated MYC-Mps1 constructs were resolved by SDS-PAGE and immunoblotted as shown.

(D) Cells were treated as in (C), enriched in mitosis with nocodazole, and immunoblotted as indicated.

See also Figures S4 and S5.

kinase assays (Figure 4A). Interestingly, immunoprecipitated, clustered Mps1-Δ40–49 still exhibited reduced activity, whereas that of a distinct deletion mutant (Mps1-Δ19–29) was comparable to Mps1-WT, suggesting that amino acids 40–49 are required for Mps1 activation. The observation that clustering restores full kinase activity to Mps1-ΔNTE and Mps1-ΔTPR, but not Mps1-Δ40–49, strongly suggests that residues 40–49 are required to modulate Mps1 autoinhibition by the NTE itself. To test this idea directly, we generated double mutants of the NTE, combining the 40–49 deletion with other NTE mutants, namely Δ19–29, Δ7, and 5A, and assessed autophosphorylation in cell extracts. The Mps1 Δ40–49/5A double mutant exhibited autophosphorylation to levels comparable with that of Mps1-WT (Figure 4B). The activity of two other double mutants, Mps1-Δ40–49/Δ7 and Mps1-Δ40–49/Δ19–29, remained unchanged compared to Mps1-Δ40–49.

The 40–49 region harbors several potential phosphorylation sites, including residues T45, T46, and the bona fide autophosphorylation site S49 (Figure 3A) [46], raising the possibility that autoinhibition may be a result of the loss of phosphorylation in this region. Collective mutation of all three sites to alanine (Mps1-3A) resulted in a marked decrease of Mps1 phosphorylation at T686, which was rescued by a triple phosphomimetic Mps1-3E mutant (Figure 4C, top 3 panels). These data suggest that phosphorylation of a short autoinhibitory motif in the NTE is required for full activation of Mps1, likely through release of an NTE-specific autoinhibitory segment.

The unexpected finding that phosphorylation in the Mps1 NTE is required for efficient Mps1 autophosphorylation and catalytic activation suggested that NTE phosphorylation might occur prior to maximal activation segment phosphorylation. To evaluate this idea more closely, we sought to determine the phosphorylation of the NTE in the hypoactive Mps1-Δ40–49 mutant. We generated a phosphospecific antibody against T33 (Figure S4), an Mps1 autophosphorylation site [45, 47, 48], and one of the residues implicated in driving relief of autoinhibition by the NTE. We found that Mps1-3A and Mps1-3E exhibited T33 phosphorylation that was equivalent to Mps1-WT, whereas neither Mps1-ΔNTE nor Mps1-KD was phosphorylated (Figure 4C, bottom panel), confirming that T33 is indeed an Mps1 autophosphorylation site. Consistently, Mps1-Δ40–49 and Mps1-WT exhibited comparable phosphorylation at T33 despite a decrease in T686 and T676 phosphorylation compared to Mps1-WT (Figure 4D). Phosphorylation of all mutants at S821 was comparable between Mps1 proteins (Figures 4C and 4D). These observations demonstrate that NTE T33 phosphorylation, in contrast to activation segment phosphorylation, is insensitive to the NTE autoinhibitory "module".

We next sought to better understand the initial steps involved in Mps1 activation. Co-transfection of differentially tagged Mps1 WT and KD resulted in phosphorylation of the KD mutant at both T686 and T33, indicating that NTE phosphorylation occurs in *trans* (Figure S5A). In addition, we tested NTE phosphorylation by Plk1, which shares a similar substrate consensus as Mps1 and has been implicated in Mps1 activation [6, 7]. We established that the NTE is a poor substrate for Plk1, at least *in vitro*, whereas it was efficiently phosphorylated by Mps1 (Figures S5B–S5E); residual phosphorylation is likely due to other target Mps1 sites in the NTE, TPR, or both. Finally, Plk1 inhibition

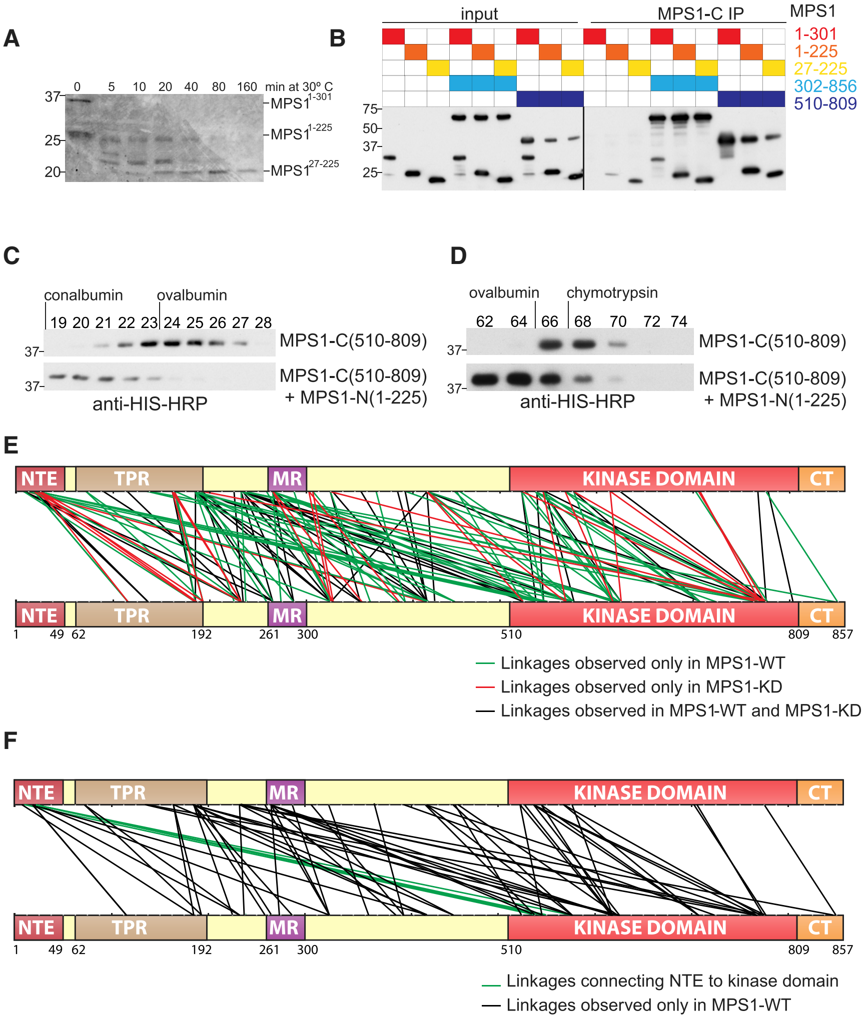

Figure 5. The Mps1 NTE Interacts Directly with the Kinase Domain

(A) A bacterially expressed, 6×HIS-tagged N-terminal fragment of Mps1 (residues 1–301) was subjected to limited proteolysis, resolved by SDS-PAGE, and identified by mass spectrometry; n = 2.

(B) Mps1 N- and C-terminal fragments were mixed, immunoprecipitated with an antibody specific for Mps1's C terminus, resolved by SDS-PAGE, and detected using an anti-His HRP-conjugated antibody; n > 3.

(C) Mps1-C (510–809) alone or mixed with Mps1-N (1–225) was subjected to velocity sedimentation on a 5%–20% glycerol gradient. Fractions were resolved by SDS-PAGE and detected as in (B). Sedimentation peaks of conalbumin and ovalbumin are shown; n = 2.

(D) Elution profile of Mps1-C and Mps1-C + Mps1-N on a Superdex 200 size-exclusion column. Elution peaks of ovalbumin and chymotrypsin are indicated; n = 2.

(E) Mps1-WT and Mps1-KD cross-linkage map. Black, linkages in both Mps1-WT and Mps1-KD; green, Mps1-WT linkages; red, Mps1-KD linkages.

(F) Mps1-WT-specific linkages; NTE-kinase links are marked in green.

See also Tables S1 and S2.

resulted in a consistent decrease of Mps1 T686 phosphorylation in both Mps1-WT- and Mps1-Δ40–49-expressing cells, indicating that Plk1 contributes to Mps1 activation independently of the NTE (Figure S5F).

The Mps1 NTE Interacts Directly with the Kinase Domain

Intermolecular NTE phosphorylation by Mps1 requires a topology in which sites proximal to the NTE interact with the active Mps1 in order to promote phosphorylation. We evaluated such a mechanism *in vitro* by employing stable, 6×His-tagged recombinant Mps1 fragments that were isolated after limited proteolysis (Figure 5A). Mps1 N-terminal fragments (residues 1–301, 1–225, and 27–225) were incubated with recombinant 6×His-tagged C-terminal Mps1 fragments (residues 302–859 and 510–809) and then immunoprecipitated with an Mps1 C terminus antibody. All N-terminal Mps1 fragments interacted directly with both C-terminal fragments of Mps1 (Figure 5B). Next, we used velocity sedimentation to determine complex formation between N- and C-terminal Mps1 fragments (Figure 5C). After co-incubation with Mps1-N(1–225), Mps1-C(510–809) displayed a shifted sedimentation profile, indicating efficient complex formation. Finally, we tested association between Mps1-N(1–225) and Mps1-C(510–809) fragments by analytical size-exclusion chromatography, which confirmed direct complex formation between the N and C termini of Mps1 (Figure 5D).

To fine-map intramolecular Mps1 interactions, and further explore the dependence of these interactions on Mps1 activity, we employed chemical cross-linking and mass spectrometry [49]. Glutathione S-transferase (GST)-Mps1-WT and KD purified to homogeneity from insect cells were individually cross-linked with bis(sulfosuccinimidyl)suberate (BS3) prior to peptide identification by mass spectrometry (Figures 5E and 5F; see also Tables S1 and S2). Importantly, we established that the chemical cross-linking map of Mps1-WT and Mps1-KD varied greatly with only a small number of shared cross-links and with Mps1-WT forming a far greater number of unique cross-links than Mps1-KD (Figure 5E). This suggests the existence of significant conformational differences between catalytically active (and highly phosphorylated) and inactive full-length Mps1 polypeptides [50]. Interestingly, interactions formed between the NTE and kinase domain of Mps1 were only identified in Mps1-WT and not Mps1-KD cross-links, suggesting that Mps1 autophosphorylation and activation is coincident with direct interactions between its N and C termini.

Mps1 NTE-Mediated Relief of Autoinhibition Is Required for SAC Activity, but Not Chromosome Segregation Fidelity

To explore the cellular consequences of NTE-mediated Mps1 autoinhibition on critical mitotic functions of Mps1, we generated

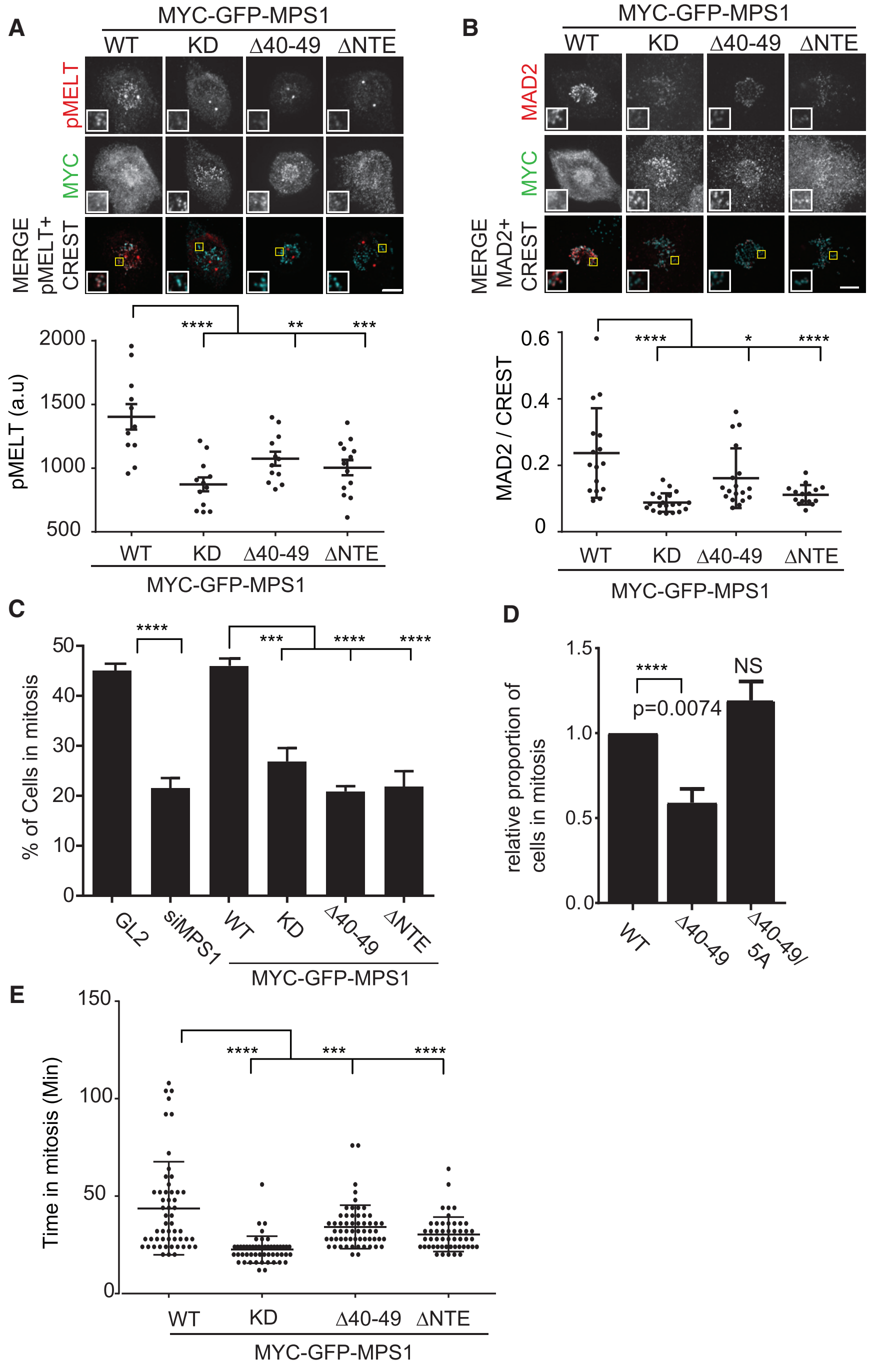

Figure 6. Relief of NTE-Mediated Autoinhibition Is Required for Activity

(A and B) Stable HeLa T-rex lines expressing MYC-GFP-Mps1-WT and mutants were treated with siMps1, synchronized in mitosis, fixed, and immunostained as indicated. The scale bar represents 5 μm. Quantification of pMELT (A) and Mad2 (B) intensities is shown. *p < 0.05; **p < 0.01; ***p < 0.001; ****p < 0.0001.

(C and D) Cells were treated as in (A) but fixed after overnight nocodazole treatment and stained with the indicated antibodies. The mitotic index is shown; n = 3. Cells counted ≥100 per cell lines per experiment.

(E) Time-lapse microscopy of cells expressing indicated constructs. Mitotic timing is quantified. ≥50 expressing cells were counted for each condition. n = 2. ***p < 0.001; ****p < 0.0001.

See also Figure S6 and Movies S1, S2, S3, and S4.

a panel of HeLa T-Rex stable cell lines expressing Mps1 NTE mutants (Figures S6A–S6D) and tested the effect of various NTE deletions and mutations on SAC dynamics, mitotic timing, and chromosome congression in cells simultaneously depleted of endogenous Mps1. As a first test for SAC signaling, we examined MELT motif phosphorylation. Mps1-WT-expressing cells demonstrated robust pMELT signals, whereas Mps1-Δ40–49- and ΔNTE-expressing cells exhibited a significant decrease in MELT motif phosphorylation, indicative of attenuated SAC signals (Figure 6A). MELT phosphorylation initiates the signaling cascade that ultimately results in the accumulation of MAD2 at kinetochores. We therefore determined the levels of O-MAD2 recruited to kinetochores, confirming that expression of Mps1-Δ40–49 significantly reduced O-MAD2 recruitment (Figure 6B) [51]. Finally, we measured mitotic arrest in the presence of nocodazole and found that Mps1-Δ40–49-expressing cells exhibited a significantly lower mitotic index when compared to Mps1-WT (Figure 6C). Importantly, in a cell line expressing Mps1-Δ40–49/5A, in which Mps1 activation loop phosphorylation was recovered (Figure 4B), the SAC arrest defect was also rescued (Figures 6D, S6C, and S6D). We next used live-cell imaging to determine the duration of mitosis (defined by the time interval between nuclear envelope breakdown and anaphase onset) in our stable cell lines. Mitotic timing was markedly decreased from an average of 40.8 ± 2.09 min for siGL2-treated cells to 26.32 ± 0.94 min (p < 0.0001 one-way ANOVA) in Mps1-depleted cells in agreement with previous literature (Figure S6E) [25, 26, 37, 52]. MYC-GFP Mps1 WT rescued mitotic timing (43.78 ± 3.25 min), whereas Mps1-KD and Mps1-ΔNTE did not, with an average duration of 22.51 ± 0.97 min and 30.40 ± 1.25 min, respectively (p < 0.0001; ANOVA; Figure 6E). Although Mps1-Δ40–49-expressing cells completed mitosis at a significantly faster rate than Mps1-WT-expressing cells, this effect was not as pronounced as that observed with the other NTE mutants (34.21 ± 1.46 min; p < 0.001; ANOVA; see Movies S1, S2, S3, and S4). Thus, the 40–49 NTE motif is required for full SAC function in addition to efficient mitotic timing.

Alongside SAC signaling, Mps1 kinase activity also regulates kinetochore-microtubule interactions [9, 25–28, 46]. To

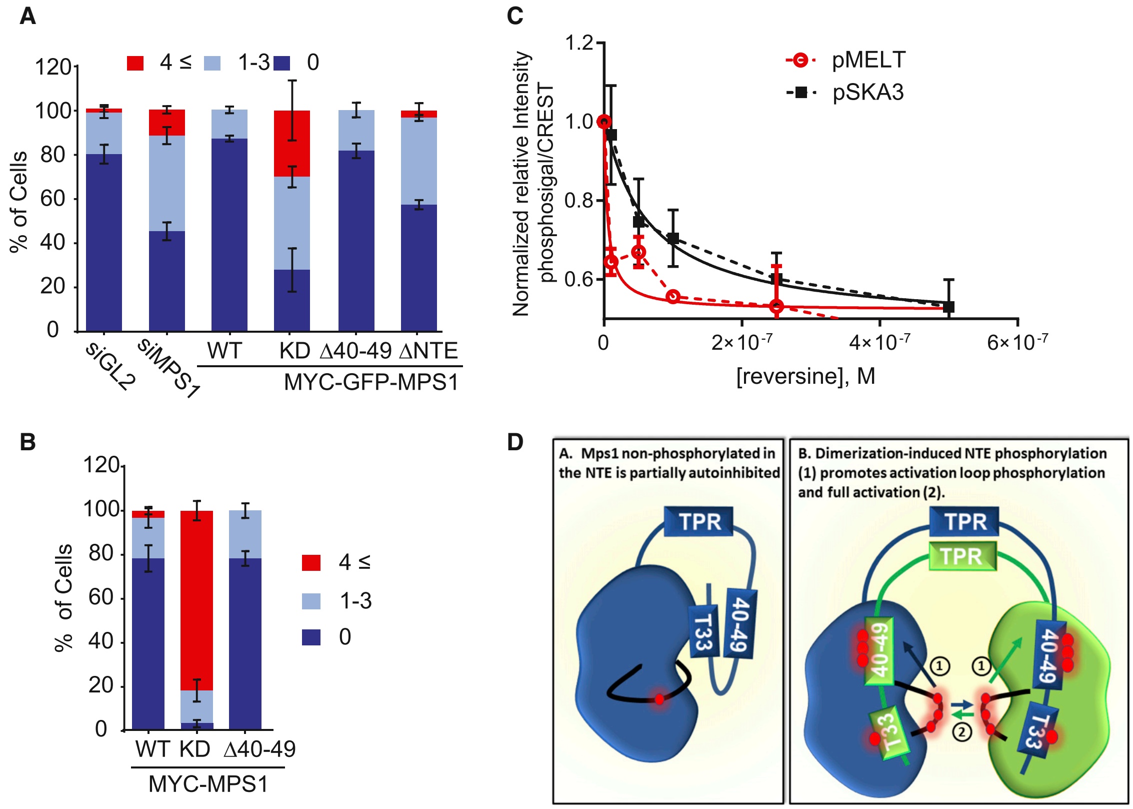

Figure 7. Differential Dephosphorylation Kinetics of Mps1 Substrates after Chemical Inhibition

(A) Stable HeLa T-rex lines expressing MYC-GFP-Mps1-WT or mutants were treated with siMps1 and synchronized in mitosis before fixation and immunostaining as shown. Lagging chromosomes in anaphase cells were determined by CREST staining. n = 3, and ≥30 anaphases were counted for each condition.

(B) Cells were treated as in (A) with the addition of nocodazole (7.5 ng/mL).

(C) Cells were treated with the indicated doses of reversine for 5 min before fixation and immunofluorescence as indicated.

(D) Model of NTE phosphorylation and Mps1 activation.

See also Figure S7.

examine the role of the NTE-mediated partial autoinhibition in this process, we evaluated the extent of lagging chromosomes in anaphase (Figure 7A). This phenotype was most pronounced in cells expressing Mps1-KD, with approximately 60% of the cells displaying lagging chromosomes. Cells expressing Mps1-ΔNTE displayed lagging chromosomes in approximately 43% of cells, respectively ($p < 0.001$; 2-way ANOVA), whereas Mps1-Δ40–49-expressing cells showed no apparent increase in lagging chromosomes when compared to Mps1-WT ($p > 0.9999$; 2-way ANOVA), even in the presence of low doses of nocodazole to induce spindle stress (Figure 7B). To understand why Mps1 Δ40–49 only attenuated the SAC functions of Mps1, we examined the effect of Mps1 partial inhibition on specific Mps1 substrates involved in supporting the SAC (pMELT) and chromosome congression (SKA3 pS34) [46]. In response to increasing doses of reversine, MELT motif phosphorylation was significantly more sensitive to Mps1 inhibition than SKA pS34 (Figure 7C). This is in agreement with very recent observations demonstrating a preference for pThr over pSer residues by PP2A upon mitotic exit [53] and potentially explains why the partial inhibition of Mps1-Δ40–49 impinges more strongly on the SAC rather than chromosome segregation.

DISCUSSION

Mps1 lies at, or near, the apex of the SAC-signaling cascade, and its localization at kinetochores serves an important role in increasing the local concentrations of Mps1 at its site of action, which in turn promotes Mps1 catalytic activity (Figure 2). We confirm that the Mps1 N-terminal region contributes to the SAC by mediating kinetochore docking of Mps1 (Figures 1A and 1B) [29, 31, 32, 35]. Moreover, the NTE also contributes to the SAC via an additional localization-independent mechanism: autoinhibition of Mps1 kinase activity. Autoinhibition is a common safety feature built into kinase regulation that prevents premature autophosphorylation until triggered by an external stimulus [54]. In the case of Mps1, we suggest that autoinhibition might be necessary to ensure no basal SAC signaling in the absence of improper microtubule attachments of sister chromatids.

In this study, we demonstrate that the NTE participates in Mps1 autoinhibition, leading to attenuation of activation loop phosphorylation. We further establish that autophosphorylation in the NTE is important to release this autoinhibition, which subsequently permits activation of Mps1 through maximal phosphorylation in the activation segment. In its partially

autoinhibited form, residual Mps1 activity does not support a full SAC response but appears to be sufficient to promote productive microtubule attachments, possibly as a result of the differential sensitivity of substrates to local dephosphorylation (Figure 7C). Our data are thus consistent with a model where Mps1 activation is a complex, multistep process that requires initial dimerization, leading to allosteric stabilization of a partially active, "prone-to-autophosphorylate" transient Mps1 conformer prior to trans-autophosphorylation at multiple sites in the Mps1 NTE residing between residues 30 and 50 [55]. This mechanistic switch then releases a partial NTE-specific autoinhibition, enabling activation segment phosphorylation at T676 and T686 (Figure 7D), which are known to be associated with kinase activation [10, 11, 56, 57]. An attractive model is that NTE phosphorylation and association with the kinase domain may result in a disorder-to-order conformational transition in this region, allowing the release of autoinhibition and promotion of catalytic autoactivation. Importantly, the effect of autoinhibition only occurs in the context of the full-length Mps1 molecule. In the absence of the NTE or the TPR, the decrease in kinase activity is a result of the loss of efficient Mps1 kinetochore docking and clustering (Figures 2 and 3B) [29–31]. Considering that T33 is one of the residues whose mutation rescues kinase activity in the Mps1-Δ40–49 mutant, its mechanism of phosphorylation is insensitive to the partial autoinhibition imposed by the NTE (Figure 4). To the best of our knowledge, this is the first example of a graded response to catalytic autoinhibition between individual autophosphorylation sites in Mps1 or any other mitotic kinase.

How might NTE phosphorylation relieve catalytic autoinhibition? Our *in vitro* binding and cross-linking experiments revealed unique interactions between the NTE of Mps1 and its kinase domain that are absent in Mps1-KD, which lacks autophosphorylation [50]. This is in agreement with the idea that NTE phosphorylation is Mps1-dependent and strongly supports our model that NTE phosphorylation is required for efficient Mps1 activation at levels that fully support mitotic functions. Mapping the kinase domain residues identified in the cross-links with the NTE onto the Mps1 kinase structure suggests that the NTE region interacts with the N-terminal lobe at the residues V^{539}FQVLNEKK^{547} and Q^{579}QHSDKIIR^{587}. Our crystal structure of the Mps1 catalytic domain in an inhibited conformation [56] shows that residues in the Q^{579}QHSDKIIR^{587} region form part of the αC helix, a structural feature critical to kinase activity, and are in close spatial proximity (5 Å or less) to the activation segment and catalytic loops (Figure S7). We thus anticipate that the Mps1 NTE and kinase domain interact to trigger long-range conformational changes that fine-tune phosphorylation of Mps1 substrates and the maintenance of high kinase activity. Structural studies of the full-length active and inactive Mps1 will be required to further test our models and reveal the precise mechanism through which NTE phosphorylation promotes activation loop phosphorylation in Mps1. However, our study provides a mechanistic framework for understanding how Mps1 catalytic activity is autoinhibited, primed, and then generated and advances our understanding of the fine-tuning mechanisms by which Mps1 exerts control over the SAC in human cells.

STAR★METHODS

Detailed methods are provided in the online version of this paper and include the following:

- KEY RESOURCES TABLE
- CONTACT FOR REAGENT AND RESOURCE SHARING
- EXPERIMENTAL MODEL AND SUBJECT DETAILS
  - Cell lines
  - Drug treatments and transfections
- METHOD DETAILS
  - Protein lysis and immunoprecipitation
  - *In vitro* kinase assays
  - Protein production and biochemistry
  - Protein-binding assays
  - Cross-linking
  - Sample preparation for mass spectrometry
  - Mass spectrometry
  - Database searching
  - Immunofluorescence
  - Microscopy and live cell imaging
- QUANTIFICATION AND STATISTICAL ANALYSIS
- DATA AND SOFTWARE AVAILABILITY

SUPPLEMENTAL INFORMATION

Supplemental Information includes seven figures, two tables, and four movies and can be found with this article online at https://doi.org/10.1016/j.cub.2018.02.002.

ACKNOWLEDGMENTS

This work was supported by operating funds to S.E. from the Cancer Research Society (grant no. 19140) and the Canadian Institutes for Health Research (no. 376557). S.E. holds an FRQS salary award. J.M. is supported by a grant from the NIH (K99CA212290). P.V.J. was supported by NIH grants R01GM094972 and P30CA008748. H.B. was supported by a Darwin Trust of Edinburgh Ph.D. Studentship. Work in the W.C.E. lab was supported by a Wellcome Principal Research Fellowship (107022) and grant to the Wellcome Centre for Cell Biology (203148).

AUTHOR CONTRIBUTIONS

Methodology, G.C., J.M., P.T., L.G.B., I.A., C.G., L.M., H.B., D.P.B., S.S., V.M.B.-G., and S.E.; Conception, G.C., J.M., P.V.J., W.C.E., and S.E.; Phosphospecific Antibodies, P.A.E. and S.E.; Writing and Editing, G.C. and S.E. with help from V.M.B.-G., P.A.E., J.M., P.V.J., and W.C.E.

DECLARATION OF INTERESTS

The authors declare no competing interests.

Received: December 8, 2017
Revised: January 15, 2018
Accepted: February 1, 2018
Published: March 1, 2018

REFERENCES

1. Faesen, A.C., Thanasoula, M., Maffini, S., Breit, C., Müller, F., van Gerwen, S., Bange, T., and Musacchio, A. (2017). Basis of catalytic assembly of the mitotic checkpoint complex. Nature *542*, 498–502.
2. Musacchio, A. (2015). The molecular biology of spindle assembly checkpoint signaling dynamics. Curr. Biol. *25*, R1002–R1018.

3. Sacristan, C., and Kops, G.J. (2015). Joined at the hip: kinetochores, microtubules, and spindle assembly checkpoint signaling. Trends Cell Biol. *25*, 21–28.
4. Musacchio, A., and Desai, A. (2017). A molecular view of kinetochore assembly and function. Biology (Basel) *6*, 5.
5. Etemad, B., and Kops, G.J. (2016). Attachment issues: kinetochore transformations and spindle checkpoint silencing. Curr. Opin. Cell Biol. *39*, 101–108.
6. von Schubert, C., Cubizolles, F., Bracher, J.M., Sliedrecht, T., Kops, G.J.P.L., and Nigg, E.A. (2015). Plk1 and Mps1 cooperatively regulate the spindle assembly checkpoint in human cells. Cell Rep. *12*, 66–78.
7. Espeut, J., Lara-Gonzalez, P., Sassine, M., Shiau, A.K., Desai, A., and Abrieu, A. (2015). Natural loss of Mps1 kinase in nematodes uncovers a role for polo-like kinase 1 in spindle checkpoint initiation. Cell Rep. *12*, 58–65.
8. Liu, X., and Winey, M. (2012). The MPS1 family of protein kinases. Annu. Rev. Biochem. *81*, 561–585.
9. Hewitt, L., Tighe, A., Santaguida, S., White, A.M., Jones, C.D., Musacchio, A., Green, S., and Taylor, S.S. (2010). Sustained Mps1 activity is required in mitosis to recruit O-Mad2 to the Mad1-C-Mad2 core complex. J. Cell Biol. *190*, 25–34.
10. Mattison, C.P., Old, W.M., Steiner, E., Huneycutt, B.J., Resing, K.A., Ahn, N.G., and Winey, M. (2007). Mps1 activation loop autophosphorylation enhances kinase activity. J. Biol. Chem. *282*, 30553–30561.
11. Tyler, R.K., Chu, M.L., Johnson, H., McKenzie, E.A., Gaskell, S.J., and Eyers, P.A. (2009). Phosphoregulation of human Mps1 kinase. Biochem. J. *417*, 173–181.
12. Vleugel, M., Omerzu, M., Groenewold, V., Hadders, M.A., Lens, S.M., and Kops, G.J. (2015). Sequential multisite phospho-regulation of KNL1-BUB3 interfaces at mitotic kinetochores. Mol. Cell *57*, 824–835.
13. Krenn, V., Overlack, K., Primorac, I., van Gerwen, S., and Musacchio, A. (2014). KI motifs of human Knl1 enhance assembly of comprehensive spindle checkpoint complexes around MELT repeats. Curr. Biol. *24*, 29–39.
14. Zhang, G., Lischetti, T., and Nilsson, J. (2014). A minimal number of MELT repeats supports all the functions of KNL1 in chromosome segregation. J. Cell Sci. *127*, 871–884.
15. Primorac, I., Weir, J.R., Chiroli, E., Gross, F., Hoffmann, I., van Gerwen, S., Ciliberto, A., and Musacchio, A. (2013). Bub3 reads phosphorylated MELT repeats to promote spindle assembly checkpoint signaling. eLife *2*, e01030.
16. Vleugel, M., Tromer, E., Omerzu, M., Groenewold, V., Nijenhuis, W., Snel, B., and Kops, G.J. (2013). Arrayed BUB recruitment modules in the kinetochore scaffold KNL1 promote accurate chromosome segregation. J. Cell Biol. *203*, 943–955.
17. London, N., Ceto, S., Ranish, J.A., and Biggins, S. (2012). Phosphoregulation of Spc105 by Mps1 and PP1 regulates Bub1 localization to kinetochores. Curr. Biol. *22*, 900–906.
18. Shepperd, L.A., Meadows, J.C., Sochaj, A.M., Lancaster, T.C., Zou, J., Buttrick, G.J., Rappsilber, J., Hardwick, K.G., and Millar, J.B. (2012). Phosphodependent recruitment of Bub1 and Bub3 to Spc7/KNL1 by Mph1 kinase maintains the spindle checkpoint. Curr. Biol. *22*, 891–899.
19. Yamagishi, Y., Yang, C.H., Tanno, Y., and Watanabe, Y. (2012). MPS1/Mph1 phosphorylates the kinetochore protein KNL1/Spc7 to recruit SAC components. Nat. Cell Biol. *14*, 746–752.
20. Ji, Z., Gao, H., Jia, L., Li, B., and Yu, H. (2017). A sequential multi-target Mps1 phosphorylation cascade promotes spindle checkpoint signaling. eLife *6*, e22513.
21. London, N., and Biggins, S. (2014). Mad1 kinetochore recruitment by Mps1-mediated phosphorylation of Bub1 signals the spindle checkpoint. Genes Dev. *28*, 140–152.
22. Kim, S., Sun, H., Tomchick, D.R., Yu, H., and Luo, X. (2012). Structure of human Mad1 C-terminal domain reveals its involvement in kinetochore targeting. Proc. Natl. Acad. Sci. USA *109*, 6549–6554.
23. De Antoni, A., Pearson, C.G., Cimini, D., Canman, J.C., Sala, V., Nezi, L., Mapelli, M., Sironi, L., Faretta, M., Salmon, E.D., and Musacchio, A. (2005). The Mad1/Mad2 complex as a template for Mad2 activation in the spindle assembly checkpoint. Curr. Biol. *15*, 214–225.
24. Tipton, A.R., Ji, W., Sturt-Gillespie, B., Bekier, M.E., 2nd, Wang, K., Taylor, W.R., and Liu, S.T. (2013). Monopolar spindle 1 (MPS1) kinase promotes production of closed MAD2 (C-MAD2) conformer and assembly of the mitotic checkpoint complex. J. Biol. Chem. *288*, 35149–35158.
25. Jelluma, N., Brenkman, A.B., van den Broek, N.J., Cruijsen, C.W., van Osch, M.H., Lens, S.M., Medema, R.H., and Kops, G.J. (2008). Mps1 phosphorylates Borealin to control Aurora B activity and chromosome alignment. Cell *132*, 233–246.
26. Tighe, A., Staples, O., and Taylor, S. (2008). Mps1 kinase activity restrains anaphase during an unperturbed mitosis and targets Mad2 to kinetochores. J. Cell Biol. *181*, 893–901.
27. Maciejowski, J., George, K.A., Terret, M.E., Zhang, C., Shokat, K.M., and Jallepalli, P.V. (2010). Mps1 directs the assembly of Cdc20 inhibitory complexes during interphase and mitosis to control M phase timing and spindle checkpoint signaling. J. Cell Biol. *190*, 89–100.
28. Santaguida, S., Tighe, A., D’Alise, A.M., Taylor, S.S., and Musacchio, A. (2010). Dissecting the role of MPS1 in chromosome biorientation and the spindle checkpoint through the small molecule inhibitor reversine. J. Cell Biol. *190*, 73–87.
29. Thebault, P., Chirgadze, D.Y., Dou, Z., Blundell, T.L., Elowe, S., and Bolanos-Garcia, V.M. (2012). Structural and functional insights into the role of the N-terminal Mps1 TPR domain in the SAC (spindle assembly checkpoint). Biochem. J. *448*, 321–328.
30. Lee, S., Thebault, P., Freschi, L., Beaufils, S., Blundell, T.L., Landry, C.R., Bolanos-Garcia, V.M., and Elowe, S. (2012). Characterization of spindle checkpoint kinase Mps1 reveals domain with functional and structural similarities to tetratricopeptide repeat motifs of Bub1 and BubR1 checkpoint kinases. J. Biol. Chem. *287*, 5988–6001.
31. Nijenhuis, W., von Castelmur, E., Littler, D., De Marco, V., Tromer, E., Vleugel, M., van Osch, M.H., Snel, B., Perrakis, A., and Kops, G.J. (2013). A TPR domain-containing N-terminal module of MPS1 is required for its kinetochore localization by Aurora B. J. Cell Biol. *201*, 217–231.
32. Hiruma, Y., Sacristan, C., Pachis, S.T., Adamopoulos, A., Kuijt, T., Ubbink, M., von Castelmur, E., Perrakis, A., and Kops, G.J. (2015). CELL DIVISION CYCLE. Competition between MPS1 and microtubules at kinetochores regulates spindle checkpoint signaling. Science *348*, 1264–1267.
33. Marquardt, J.R., Perkins, J.L., Beuoy, K.J., and Fisk, H.A. (2016). Modular elements of the TPR domain in the Mps1 N terminus differentially target Mps1 to the centrosome and kinetochore. Proc. Natl. Acad. Sci. USA *113*, 7828–7833.
34. Majumder, S., Slabodnick, M., Pike, A., Marquardt, J., and Fisk, H.A. (2012). VDAC3 regulates centriole assembly by targeting Mps1 to centrosomes. Cell Cycle *11*, 3666–3678.
35. Ji, Z., Gao, H., and Yu, H. (2015). CELL DIVISION CYCLE. Kinetochore attachment sensed by competitive Mps1 and microtubule binding to Ndc80C. Science *348*, 1260–1264.
36. Aravamudhan, P., Goldfarb, A.A., and Joglekar, A.P. (2015). The kinetochore encodes a mechanical switch to disrupt spindle assembly checkpoint signalling. Nat. Cell Biol. *17*, 868–879.
37. Dou, Z., Liu, X., Wang, W., Zhu, T., Wang, X., Xu, L., Abrieu, A., Fu, C., Hill, D.L., and Yao, X. (2015). Dynamic localization of Mps1 kinase to kinetochores is essential for accurate spindle microtubule attachment. Proc. Natl. Acad. Sci. USA *112*, E4546–E4555.
38. Chmielewska, A.E., Tang, N.H., and Toda, T. (2016). The hairpin region of Ndc80 is important for the kinetochore recruitment of Mph1/MPS1 in fission yeast. Cell Cycle *15*, 740–747.
39. Heinrich, S., Windecker, H., Hustedt, N., and Hauf, S. (2012). Mph1 kinetochore localization is crucial and upstream in the hierarchy of spindle assembly checkpoint protein recruitment to kinetochores. J. Cell Sci. *125*, 4720–4727.

40. Araki, Y., Gombos, L., Migueleti, S.P., Sivashanmugam, L., Antony, C., and Schiebel, E. (2010). N-terminal regions of Mps1 kinase determine functional bifurcation. J. Cell Biol. *189*, 41–56.

41. Jelluma, N., Dansen, T.B., Sliedrecht, T., Kwiatkowski, N.P., and Kops, G.J. (2010). Release of Mps1 from kinetochores is crucial for timely anaphase onset. J. Cell Biol. *191*, 281–290.

42. Kang, J., Chen, Y., Zhao, Y., and Yu, H. (2007). Autophosphorylation-dependent activation of human Mps1 is required for the spindle checkpoint. Proc. Natl. Acad. Sci. USA *104*, 20232–20237.

43. Wang, W., Yang, Y., Gao, Y., Xu, Q., Wang, F., Zhu, S., Old, W., Resing, K., Ahn, N., Lei, M., and Liu, X. (2009). Structural and mechanistic insights into Mps1 kinase activation. J. Cell. Mol. Med. *13* (8B), 1679–1694.

44. Janicki, S.M., Tsukamoto, T., Salghetti, S.E., Tansey, W.P., Sachidanandam, R., Prasanth, K.V., Ried, T., Shav-Tal, Y., Bertrand, E., Singer, R.H., and Spector, D.L. (2004). From silencing to gene expression: real-time analysis in single cells. Cell *116*, 683–698.

45. Dou, Z., von Schubert, C., Körner, R., Santamaria, A., Elowe, S., and Nigg, E.A. (2011). Quantitative mass spectrometry analysis reveals similar substrate consensus motif for human Mps1 kinase and Plk1. PLoS ONE *6*, e18793.

46. Maciejowski, J., Drechsler, H., Grundner-Culemann, K., Ballister, E.R., Rodriguez-Rodriguez, J.A., Rodriguez-Bravo, V., Jones, M.J.K., Foley, E., Lampson, M.A., Daub, H., et al. (2017). Mps1 regulates kinetochore-microtubule attachment stability via the Ska complex to ensure error-free chromosome segregation. Dev. Cell *41*, 143–156.e6.

47. Xu, Q., Zhu, S., Wang, W., Zhang, X., Old, W., Ahn, N., and Liu, X. (2009). Regulation of kinetochore recruitment of two essential mitotic spindle checkpoint proteins by Mps1 phosphorylation. Mol. Biol. Cell *20*, 10–20.

48. Jelluma, N., Brenkman, A.B., McLeod, I., Yates, J.R., 3rd, Cleveland, D.W., Medema, R.H., and Kops, G.J. (2008). Chromosomal instability by inefficient Mps1 auto-activation due to a weakened mitotic checkpoint and lagging chromosomes. PLoS ONE *3*, e2415.

49. Barysz, H., Kim, J.H., Chen, Z.A., Hudson, D.F., Rappsilber, J., Gerloff, D.L., and Earnshaw, W.C. (2015). Three-dimensional topology of the SMC2/SMC4 subcomplex from chicken condensin I revealed by cross-linking and molecular modelling. Open Biol. *5*, 150005.

50. Johnson, H., Eyers, C.E., Eyers, P.A., Beynon, R.J., and Gaskell, S.J. (2009). Rigorous determination of the stoichiometry of protein phosphorylation using mass spectrometry. J. Am. Soc. Mass Spectrom. *20*, 2211–2220.

51. Fava, L.L., Kaulich, M., Nigg, E.A., and Santamaria, A. (2011). Probing the in vivo function of Mad1:C-Mad2 in the spindle assembly checkpoint. EMBO J. *30*, 3322–3336.

52. Colombo, R., Caldarelli, M., Mennecozzi, M., Giorgini, M.L., Sola, F., Cappella, P., Perrera, C., Depaolini, S.R., Rusconi, L., Cucchi, U., et al. (2010). Targeting the mitotic checkpoint for cancer therapy with NMS-P715, an inhibitor of MPS1 kinase. Cancer Res. *70*, 10255–10264.

53. Hein, J.B., Hertz, E.P.T., Garvanska, D.H., Kruse, T., and Nilsson, J. (2017). Distinct kinetics of serine and threonine dephosphorylation are essential for mitosis. Nat. Cell Biol. *19*, 1433–1440.

54. Bayliss, R., Fry, A., Haq, T., and Yeoh, S. (2012). On the molecular mechanisms of mitotic kinase activation. Open Biol. *2*, 120136.

55. Beenstock, J., Mooshayef, N., and Engelberg, D. (2016). How do protein kinases take a selfie (autophosphorylate)? Trends Biochem. Sci. *41*, 938–953.

56. Chu, M.L., Chavas, L.M., Douglas, K.T., Eyers, P.A., and Tabernero, L. (2008). Crystal structure of the catalytic domain of the mitotic checkpoint kinase Mps1 in complex with SP600125. J. Biol. Chem. *283*, 21495–21500.

57. Chu, M.L., Lang, Z., Chavas, L.M., Neres, J., Fedorova, O.S., Tabernero, L., Cherry, M., Williams, D.H., Douglas, K.T., and Eyers, P.A. (2010). Biophysical and X-ray crystallographic analysis of Mps1 kinase inhibitor complexes. Biochemistry *49*, 1689–1701.

STAR★METHODS

KEY RESOURCES TABLE

REAGENT or RESOURCE	SOURCE	IDENTIFIER
Antibodies		
anti-MYC (Clone 9E10)	Thermo Fisher Scientific	Cat# MA1-980-1MG, RRID:AB_2537627
anti Knl1 pT875	Gift of Dr. Yoshiri Watanabe, University of Tokyo	N/A
Anti-Mps1	Sigma-Aldrich	Cat# M5818, RRID:AB_261965
Anti-Mps1 pT33	This study	N/A
Anti-Mps1 pT676	Eyers Lab [11]	N/A
Anti-Mps1 pT686	Eyers Lab [11]	N/A
Anti-Mps1 pS821	Eyers Lab [11]	N/A
Anti-Mps1 C terminus	Santa Cruz Biotechnology	Cat# sc-540, RRID:AB_632567
Anti-Mad2 Clone #107-276-3	Gift of Erich Nigg [50]	N/A
Anti-α-tubulin (Clone DM1A)	Santa-Cruz Biotechnology	Cat# sc-32293, RRID:AB_628412
Anti-Cenp-C	MBL International	Cat# PD030, RRID:AB_10693556
Anti- polyHistidine-HRP peroxidase	Sigma	Cat# A7058
Anti-GFP	Sigma-Aldrich (Roche)	Cat# 11814460001, RRID:AB_390913
Ant-CREST	Immunovision	Cat# HTC-0100
Bacterial and Virus Strains		
Escherichia coli: BL21(DE3)	EMB Millipore	Cat. No. #69450
Spodoptera frugiperda: SF9	ATCC	CRL-1711, CLS Cat# 604328/p700_Sf9, RRID:CVCL_0549
Chemicals, Peptides, and Recombinant Proteins		
Nocodazole	Sigma-Aldrich	Cat# M1404-10MG
Taxol (Paclitaxel, Taxus sp)	Calbiochem	Cat# 580555
Reversine	Sigma-Aldrich	Cat# R3904-1MG
BI2536	Reagents Direct	88-Q71
Thymidine	Acros	Cat# 226740050
Hygromycin B	Wisent (MultiCell)	Cat# 450-141-XL
Tetracycline	Sigma-Aldrich	Cat# T7660-5G
Polyethylenimine	Polysciences	Cat# 23966
bis(sulfosuccinimidyl)suberate (BS3)	Thermo Fisher Scientific	Cat# 21580
Blaticidin S HCL	GIBCO	Cat# A11139-03
Mps1 T33 phosphopeptide: CKNEDL(pT)DELS	Custom synthesis, EZBiolab	N/A
Critical Commercial Assays		
Pierce BCA Protein Assay Kit	ThermoScientific	Cat# 23225
Trans-IT transfection kit	Mirus Bio	Cat# MIR2305
JetPRIME	Polyplus-transfection	Cat# 114-07
Oligofectamine	Invitrogen	Cat# 12252-011
glutathione-agarose beads (Glutathione Sepharose 4B Media)	GE Healthcare	Cat# 45-000-139
Experimental Models: Cell Lines		
Human: HeLa S3	E.Nigg Lab	ATCC Cat# CRL-7924, RRID:CVCL_0058
Human: HeLa T-REX	A.Desai Lab	N/A
Human: HEK293T	E.Nigg Lab	ATCC Cat# CRL-3216, RRID:CVCL_0063
Human: hTERT-RPE1	E.Nigg Lab	ATCC Cat# CRL-4000

(*Continued on next page*)

Continued		
REAGENT or RESOURCE	SOURCE	IDENTIFIER
Oligonucleotides		
Mps1 DsiRNA Oligos A: 5′-GUAGAUUUCCACA GGGAUUCAAGAGUA- 3′	DTI	N/A
Mps1 DsiRNA Oligos B: 5′-AUACAGUGCCAUA AGUGGUUGCUAUUU-3′	DTI	N/A
GL2i (Luciferase Gl2 siRNA) sense: 5′-UCG AAG UAU UCC GCG UAC G(dT)(dT)- 3′, anti-sense: 5′-CGU ACG CGG AAU ACU UCG A(dT)(dT)-3′	Life Technologies	N/A
Recombinant DNA		
pcDNA 3.1-3xmyc-(A)-Mps1 WT	[29, 30]	N/A
pcDNA 3.1-3xmyc-(A)-Mps1 KD	[29,30]	N/A
pcDNA 3.1-3xmyc-(A)-Mps1 ΔNTE	This study	N/A
pcDNA 3.1-3xmyc-(A)-Mps1 Δ7	This study	N/A
pcDNA 3.1-3xmyc-(A)-Mps1 5A	This study	N/A
pcDNA 3.1-3xmyc-(C)-Mis12-Mps1 ΔNTE	This study	N/A
pcDNA 5 TO/FRT-3xmyc-(C)-Mps1 WT	This study	N/A
pcDNA 5 TO/FRT-3xmyc-(C)-Mps1 KD	This study	N/A
pcDNA 5 TO/FRT-3xmyc-(C)-Mps1 Δ40-49	This study	N/A
pcDNA 5 TO/FRT-3xmyc-(C)-Mps1 Δ19/29	This study	N/A
pcDNA 5 TO/FRT-3xmyc-(C)-Mps1 ΔNTE	This study	N/A
pcDNA 5 TO/FRT-3xmyc-(C)-Mps1 40-49/5A	This study	N/A
PGEX 6P3 GST-HEC1 CH domain	This study	N/A
pcDNA 3.1-3xmyc-A-Mps1 ΔTPR	This study	N/A
pcDNA 3.1-3xmyc-(A)-FKBP-Mps1 WT	This study	N/A
pcDNA 3.1-3xmyc-(A)-FKBP-Mps1 KD	This study	N/A
pcDNA 3.1-3xmyc-(C)-FKBP-Mps1 ΔNTE	This study	N/A
pcDNA 3.1-3xmyc-(A)-LacI -Mps1WT	This study	N/A
pcDNA 3.1-3xmyc-(A)-LacI -Mps1 KD	This study	N/A
pcDNA 3.1-3xmyc-(C)-LacI -Mps1 Δ49	This study	N/A
pcDNA 3.1-3xmyc-(A)-Mps1 Δ19-29	This study	N/A
pcDNA 3.1-3xmyc-(A)-Mps1 Δ40-49	This study	N/A
pDest17-Mps1 (1-301)	This study	N/A
pDest17-Mps1 (1-225)	This study	N/A
pDest17-Mps1 (27-225)	This study	N/A
pDest17-Mps1 (302-856)	This study	N/A
pDest17-Mps1 (510-801)	This study	N/A
pDest20-Mps1-WT	This study	N/A
pDest20-Mps1-KD	This study	N/A

CONTACT FOR REAGENT AND RESOURCE SHARING

Further information and requests for resources and reagents should be directed to and will be fulfilled by the Lead Contact, Sabine Elowe (Sabine.Elowe@crchudequebec.ulaval.ca).

EXPERIMENTAL MODEL AND SUBJECT DETAILS

Cell lines

Human HeLa-S3 (Female), HeLa T-Rex (Female) and HEK293T cells were grown at 37°C with 5% CO_2 in DMEM (Hyclone) supplemented with 10% fetal bovine serum or bovine growth serum (PAA) and pen/strep (100 μg/ml, Hyclone). U2OS cells expressing a 256-copy array of the Lac operator sequences were a kind gift of David Spector (CSHL) and were maintained in the presence of 100 mg/ml of hygromycin B. HeLa Flp-in T-Rex cell line was used for generation of stable isogenic cell lines, and was a generous gift from Arshad Desai (Ludwig cancer research).

Stable cell lines were generated using Flp-In system (Life Technologies). Transfection was done with TransIT®-LT1 (Mirus Bio) reagent according to the manufacturer's instructions. The transfection reagent was mixed with the pOG44 Flp-Recombinase Expression Vector and the pCDNA5/TO/FRT-Mps1 vector with a 1:1 ratio in OptiMEM media. Selection started 48h after the transfection in a DMEM media supplemented with 10% of bovine growth serum (PAA), pen/strep (100 μg/ml, Hyclone) and in the presence of hygromycin B (200 mg/ml, MultiCell) and blasticidin (10 μg/ml, GIBCO by Life Technologies). Media was replaced 3 times a week for 3 weeks and then single colonies were picked and grown separately. Expression of the Mps1 construct was then tested by IF and WB after 24h of tetracycline treatment.

Drug treatments and transfections

Drug treatments were realized as follows: nocodazole (Sigma, as indicated, 16h), thymidine (Acros Organics, 2 mM for 16 h), reversine (Sigma, 0.5 μM, 30min), taxol (Calbiochem, 0.2 μM, 16h). For experiments with stable cell lines, 24h before harvesting cells, 1μg/ml of tetracycline was added to express the protein of interest. Plasmid transfections were performed with PEI (polyethylenimine) at a 15:2 (PEI/DNA) ratio for HEK293T cells and with TransIT® (Mirus Bio) for HeLa S3 cells according to the manufacturer's instructions. Endogenous protein depletion was carried out with DsiRNAs (IDT), using Oligofectamine(Invitrogen) for HEK293T cells and HeLa-S3 or JetPRIME® (Polyplus-transfection) for stable cell line HeLa Flp-In Trex and analyzed at 48-72h after transfection. The Mps1 and Hec1 DsiRNA targets the following sequence:

Mps1 A: 5′-GUAGAUUUCCACAGGGAUUCAAGAGUA- 3′
Mps1 B: 5′-AUACAGUGCCAUAAGUGGUUGCUAUUU-3′

Site-directed mutagenesis was performed using PCR and overlapping mutagenic primers. All constructs were verified by sequencing.

METHOD DETAILS

Protein lysis and immunoprecipitation

Lysis of cell pellets for immunoblotting and immunoprecipitation was done with RIPA lysis buffer (150 mM Tris-HCl pH 7.5, 150 mM NaCl, 10 mM NaF, 1% NP-40, 0.1% Na-deoxycholate and a protease and phosphatase inhibitor cocktail that included 20 mM β-glycerophosphate, 0.1 mM sodium vanadate, 10 mM sodium pyrophosphate, 1 mg/ml, leupeptin, 1 mg/ml, aprotinin and 1 mM AEBSF) or NP-40 lysis buffer (75 mM HEPES pH 7.5, 150mM KCl, 1.5 mM EGTA, 1.5 mM $MgCl_2$, 10% Glycerol, 0.075% NP-40 and the same protease and phosphatase inhibitor cocktail used in the RIPA buffer). For at least 30min, cells were lysed on an orbital shaker at 4°C and lysates were centrifuged at 20000xg for 10min at 4°C. The supernatant was collected and quantified using the BCA assay (Thermo Scientific) for all experiments. Equalized protein lysates were used for all experiments. Immunoprecipitations were performed with anti-MYC antibodies bound to agarose beads overnight. Beads were washed 3 times with lysis buffer and 1 time with the corresponding buffer supplemented with extra 150 mM NaCl or KCl.

In vitro kinase assays

HEK293T cells were transfected as described above and treated with nocodazole to synchronize cells in mitosis. The immunoprecipitates were given 1 final wash with Kinase Reaction Buffer (KRB: 50 mM Tris pH 7.4, 10 mM $MgCl_2$, 1 mM DTT, 100 μM Sodium-orthovanadate, 10 mM Sodium Fluoride), and then incubated with KRB supplemented with 200 μM unlabeled ATP, 5 μCi ATP (^{32}P) and MBP (1-2 μg) substrate at 30°C for 30 min with agitation. The addition of SDS–PAGE sample buffer stopped the reaction. Samples were heated for 5 min at 95°C, and resolved by SDS-PAGE for protein separation. The acrylamide gel was then stained with Coomassie Brilliant Blue, destained to remove background, then dried on Whatman paper prior to visualization by autoradiography.

Protein production and biochemistry

pGEX-6P3-Hec1 CH domain plasmid was transformed into BL21 competent cells and selected on an ampicillin-treated LB plate. One colony was picked and was seeded in a 10ml tube of LB-ampicillin, overnight at 37°C and 250 rpm. From this pre-culture, a 200ml culture was started at 37°C and 250 rpm until an O.D value of 0.7-0.9. Synthesis of the recombinant protein was started by adding 0.5 mM of IPTG (MP biomedical) for 5h at 30°C and 250 rpm. Bacteria were then centrifuged at 7700xg for 10min at 4°C and washed one time with PBS 1X and frozen. The next day, the pellet was then lysed in Lysis buffer II ([35] 25 mM Tris, 100 mM NaCl, 5% glycerol,1mM β-Mercaptoethanol, 0.1% Triton X-100, 20 mM β-glycerophosphate, 10 mM Na-pyrophosphate, 100 mM Na-Vanadate, 1μg/ml leupeptin, 1μg/ml aprotinin, 1mM AEBSF, pH8). After sonication (3 rounds of 30 s each at 10% amplitude, waiting for 0.5 s between pulses and 1min on ice between rounds) and 30min incubation on ice, the lysate was centrifuged for 10min at 4°C and 13000 rpm. The supernatant was incubated on an orbital shaker for 2h at 4°C with glutathione/Sepharose beads and then washed 2 times with lysis buffer, 1 time with TBS 1X and 1 time with GTB buffer (80 mM PIPES pH7, 2 mM $MgCl_2$, 0.5 mM EGTA). Glycerol was added to 50% (v:v) and the beads were frozen. Mps1 N and C-terminal fragments were purified from BL21(DE3) cells as hexahistidine fusions by standard nickel affinity chromatography. To obtain active Mps1, baculovirus-infected Sf9 cells were lysed by sonication in insect cell breakage buffer (100 mM Tris, pH 7.5, 20% sucrose, 4 mM EDTA, and 0.01% NP-40). GST-Mps1 was retrieved on glutathione-agarose beads (GE Healthcare), washed extensively with wash buffer (100 mM

Tris, pH 7.5, 20% sucrose, 4 mM EDTA, 500 mM KCl), and eluted in K buffer (20 mM potassium phosphate, pH 7.5, 10% glycerol, 0.5 mM EDTA, 0.5% NP-40, 25 mM glutathione, and 2 mM DTT). Eluted material was stored in single-use aliquots at −80°C. Analytical size exclusion chromatography was performed on a Superdex 200 column with 50 mM Tris, pH 7.4, 150 mM NaCl, 1 mM EDTA, 1 mM DTT as the mobile phase. For velocity sedimentation, samples were loaded onto a 5%–20% glycerol gradient (in 25 mM Tris, pH 7.5, 0.01% NP-40, and 400 mM NaCl) and centrifuged at 250,000xg for 24 hours at 4°C. Limited proteolysis was performed at 30°C while samples were mixed at 300 rpm with 15 μg of Mps1(1-301) and diluted trypsin for 160 minutes. Reactions were terminated with SDS-PAGE sample buffer and analyzed by SDS-PAGE, Coomassie Brilliant Blue staining and mass spectrometry.

Protein-binding assays

For Hec1 CH domain pull-down assays, empty beads or beads bound to GST-Hec1 CH domain were incubated for 1h with end-on mixing in GTB buffer containing 5% glycerol and 1mg/ml of BSA. Beads were washed once and recombinant Mps1 protein fragments were added to the beads in GTB buffer supplemented with 5% glycerol, 1mg/ml of BSA and 250 mM NaCl. The mix was incubated for a minimum of 2h or overnight on an orbital shaker at 4°C. After binding, beads were washed 4 times with the supplemented GTB buffer. SDS-PAGE sample buffer was added and samples were heated to 95°C for 5min amd proteins were visualized by western blotting.

Cross-linking

To find the optimal ratio of protein: cross-linker, 1 μg of Mps1 WT and 1 μg of Mps1 KD at 0.1 μg/μl in 50 mM HEPES pH7.5, 250 mM KCl, 0.05% NP-40, 1 mM DTT were incubated with a 0-, 1-, 10-, 25-, 60-fold weight excess of BS3 for 2h on ice. The reaction was quenched by addition of ammonium bicarbonate to 50 mM. The cross-linked proteins were analyzed by SDS-PAGE and NATIVE-PAGE.

For cross-linked peptides identification, 100 μg of Mps1 WT and 100 μg of Mps1 KD were cross-linked at 10:1 protein:BS3 ratio for 2h on ice and the reaction was quenched by addition of ammonium bicarbonate to 50 mM. Proteins were separated in NATIVE-PAGE and stained with Coomassie blue.

Sample preparation for mass spectrometry

Bands containing the cross-linked Mps1 dimers were excised from gel and in-gel digested following standard protocols. The cross-linked peptides were extracted from gel slices, acidified to pH 3.0 with 0.5% acetic acid and fractionated using the SCX-StageTip. High salt fractions were diluted four-fold with 0.1% TFA and desalted using C18-StageTips before MS analysis [57].

Mass spectrometry

Cross-linked peptides were analyzed on LTQ-Orbitrap Velos (Thermo Scientific) on a 180 min gradient, using CID collision energy at 35% and fragmenting the eight most intense peptide precursor ions with charge stages $z = 3$ or higher, per cycle. MS spectra were recorded at 100000 resolution, and MS/MS spectra at 7500 resolution, both in the Orbitrap.

Database searching

The MS/MS spectra peak lists were generated from the raw data files using the Quant module of MaxQuant v. 1.0.11.2 at default parameters, except for choosing 200 as ‘top MS/MS peaks per 100 Da’. Cross-linked peptide spectra were searched using the software package Xi (ERI, Edinburgh) against Mps1 sequence uploaded from SwissProt. Search parameters: MS tolerance 6 ppm, MS/MS tolerance 20 ppm, fixed modification carbamidomethyl on cysteine, variable modifications: oxidation (Met), DST/BS3-OH (Lys), DST/BS3-NH2 (Lys), the ‘Max. missed cleavages’ was set to 4. Matched spectra and cross-linked peptide candidates were returned by Xi in pairs. The highest scored matched spectra were validated manually, and to each spectral match a confidence was allocated. A high-confidence match indicates that for the longer peptide almost all, and for shorter peptides a minimum of three fragments were matched and all major peaks in the spectrum were accounted for. A low-confidence match indicates that one peptide matched essentially all observed fragments and a second peptide had up to three fragments matched with most of the peaks in spectrum explained. Reverse peptide sequences were used as a decoy search. All matches had to be highest ranking and unambiguous in the target and decoy search.

Immunofluorescence

Cells were grown on coverslips and arrested in mitosis either by nocodazole (330 nM) or by 10–12h release from thymidine block. For experiments with HeLa Flip-in T-rex cells expressing Mps1 stably, cells were grown on coverslips coated with a solution of PEI 25 μg/ml and 150 mM NaCl. Cells were fixed in PTEMF buffer (0.2% Triton X-100, 20 mM PIPES pH 6.8, 1 mM $MgCl_2$, 10 mM EGTA and 4% formaldehyde) for 10min or an Mps1-specific fixation (5min incubation with 1% formaldehyde in PBS, followed by 0.1 M glycine treatment as a quencher for 1h and 0.1% Triton X-100 for 3min to permeabilize cells) when visualizing Mps1. Before starting immunofluorescence, coverslips were incubated for at least 30 min with 3% BSA in PBS-T 0.2%. Coverslips were incubated with primary and secondary antibodies for 2h and 1h respectively at room temperature, except for Mps1 pT686, pSKA3, and Knl1 pT875 (pMELT), which were incubated at 4°C overnight in humidified chambers.

Antibodies were used at 1 μg/ml, unless otherwise indicated, as follows: anti-MYC (9E10, Thermo Scientific), anti Knl1 pT875 (1:2000, gift from Dr Yoshinori Watanabe, University of Tokyo), anti-Mps1 (1mg/ml for WB and 5 mg/ml for IF, Sigma), Rabbit

polyclonal phosphospecific antibodies against Mps1 T686, T676 and S821 [11], Rabbit polyclonal phosphospecific antibody against Mps1 T33 (1:500) was generated against phosphopeptide CKNEDL(pT)DELS, anti Mad2 (4μg/ml [50],) anti-GFP (1:500, Roche), anti-α-tubulin (DM1A, Santa Cruz Biotechnology), CENP-C (1:1000, MBL International), CREST anti-centromere serum (1:1000, ImmunoVision), and Hoechst 33342 (1μg/ml, Sigma). An antibody specific for Mps1's C terminus (Santa Cruz Biotechnology; sc-540) was used for Mps1 immunoprecipitations. Hexahistidine-fragments were detected using horseradish peroxidase-conjugated anti-histidine tag monoclonal antibody (Thermo Fisher). Dylight series secondary antibodies (Thermo Fisher) or (Jackson ImmunoResearch) were used for immunofluorescence (1:1000) and horseradish peroxidase-coupled secondary antibodies (Jackson ImmunoResearch) were used for western blotting (1:10000).

Microscopy and live cell imaging

All images were acquired by confocal microscopy on an inverted Olympus IX80 microscope equipped with a WaveFX-Borealin-SC Yokogawa spinning disc (Quorum Technologies) and an Orca Flash4.0 camera (Hamamatsu). Metamorph software (Molecular Devices) was used to perform image acquisition. Optical sections were acquired with identical exposure times for each channel within an experiment and then projected into a single picture using ImageJ (http://rsb.info.nih.gov). For image processing ImageJ or Photoshop were used and all images shown in the same figure have been identically scaled. Live cell imaging was performed on the above indicated microscopy system that is also equipped with a motorized stage (ASI) and an incubator with atmospheric CO_2 heated to 37°C. Mps1 stable cell lines were subjected to depletion of endogenous Mps1 for 72 hours, then synchronized in mitosis after a further 16h block with thymidine. Image acquisition was started 7h after release at 4min intervals. Only cells visibly expressing the GFP-tagged Mps1 were included in subsequent analysis.

QUANTIFICATION AND STATISTICAL ANALYSIS

Unless otherwise stated, all experiments were performed in triplicate. Image quantification was realized using ImageJ. For measurement of signal intensities at kinetochores, the CREST, CENP-C or MYC signal were used to generate a binary mask to include kinetochore and centromere signals. Integrated signal intensity was measured in all relevant channels and intensities indicated are values relative to CREST, CENP-C or MYC, unless otherwise stated. A minimum of ten cells was quantified per condition for all experiments involving kinetochore quantification. All statistical analysis was performed with Sigmaplot. Data are expressed as means ± SEM. The data were analyzed by ANOVA (one or two way) with Bonferroni's multiple comparison tests for determination of the significance of the differences or as otherwise indicated. Statistical significance was considered with a P value of < 0.05. The symbols * were used in the different graphics to represent the P value: *$p < 0.05$, ** $p < 0.01$, *** $p < 0.001$, **** $p < 0.0001$.

DATA AND SOFTWARE AVAILABILITY

All MS cross-links of Mps1-WT and Mps1-KD as supplied in Tables S1 and S2.

Current Biology, Volume *28*

Supplemental Information

Mps1 Phosphorylates Its N-Terminal Extension to Relieve Autoinhibition and Activate the Spindle Assembly Checkpoint

Guillaume Combes, Helena Barysz, Chantal Garand, Luciano Gama Braga, Ibrahim Alharbi, Philippe Thebault, Luc Murakami, Dominic P. Bryne, Stasa Stankovic, Patrick A. Eyers, Victor M. Bolanos-Garcia, William C. Earnshaw, John Maciejowski, Prasad V. Jallepalli, and Sabine Elowe

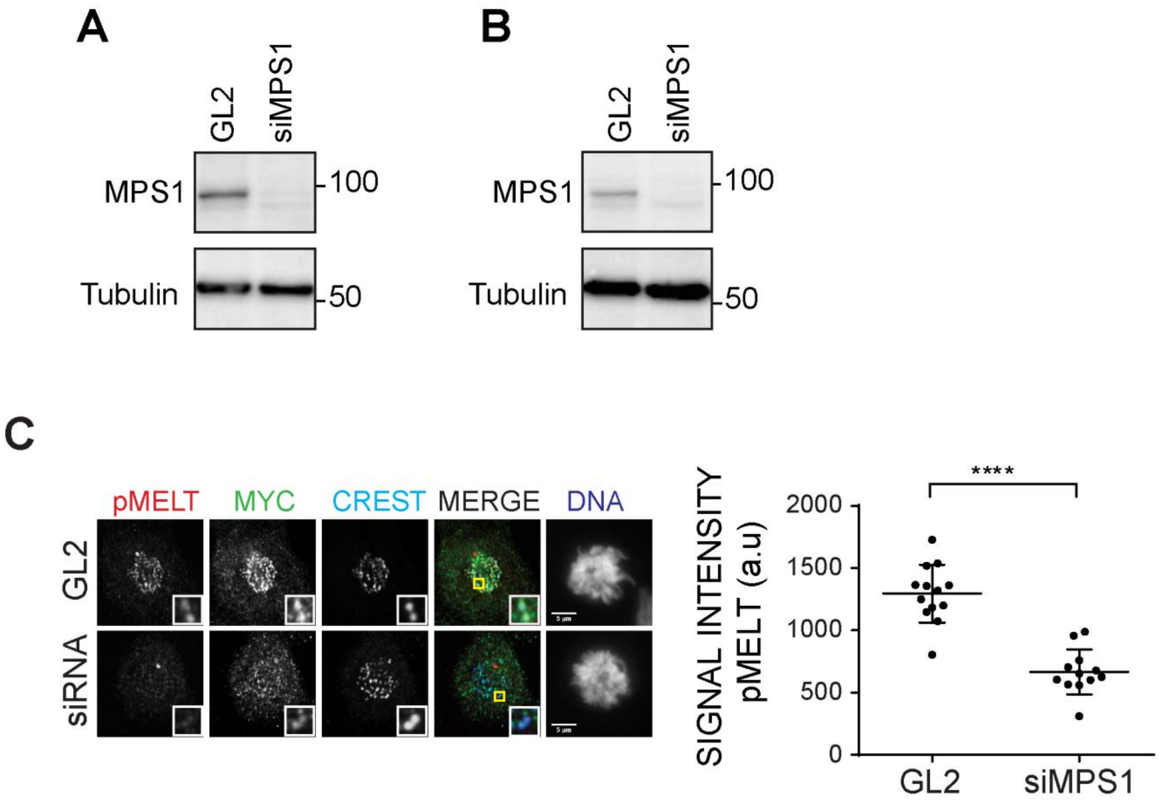

Figure S1: Verification of Mps1 siRNA efficiency, related to Figure 1.

siRNA GL2 (75nM) or siRNA Mps1-A (25nM)+Mps1-B (50nM) were transfected in either HeLa S3 **(A)** or HeLa T-Rex **(B)** cell lines for 72h before cells were lysed and cleared lysates were blotted for Mps1 (upper panels) and tubulin (lower panels). **(C)** pMELT signals in HeLa T-Rex cells depleted of endogenous Mps1. Cells were synchronized in mitosis after release from thymidine arrest before being fixed and immunostained for pMELT (Red) for, MYC1 (Green), CREST (Cyan) and Hoechst (Blue). Scale bar = 5μm. The graph shows the quantification of the relative pMELT intensity from. **** $P < 0.0001$.

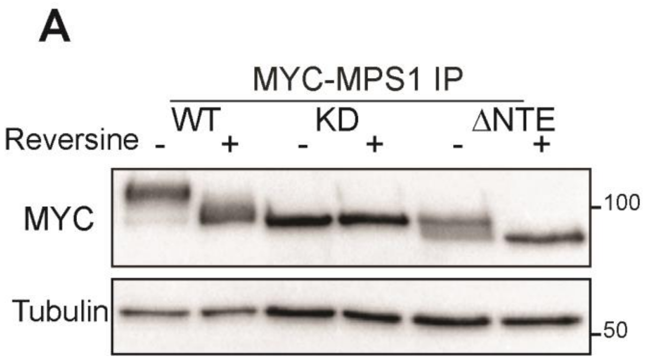

Figure S2: The NTE contributes to Mps1 mitotic hyperphosphorylation, related to Figure 1

(A) HEK 293T cells were transiently transfected with MYC-Mps1 constructs together with siMps1, and enriched in mitosis by nocodazole treatment. Cells were treated as indicated with 0.5 µM reversine 30min before harvesting. MYC (top panel) and α-tubulin (bottom panel) immunoblotting were performed on equalized lysates.

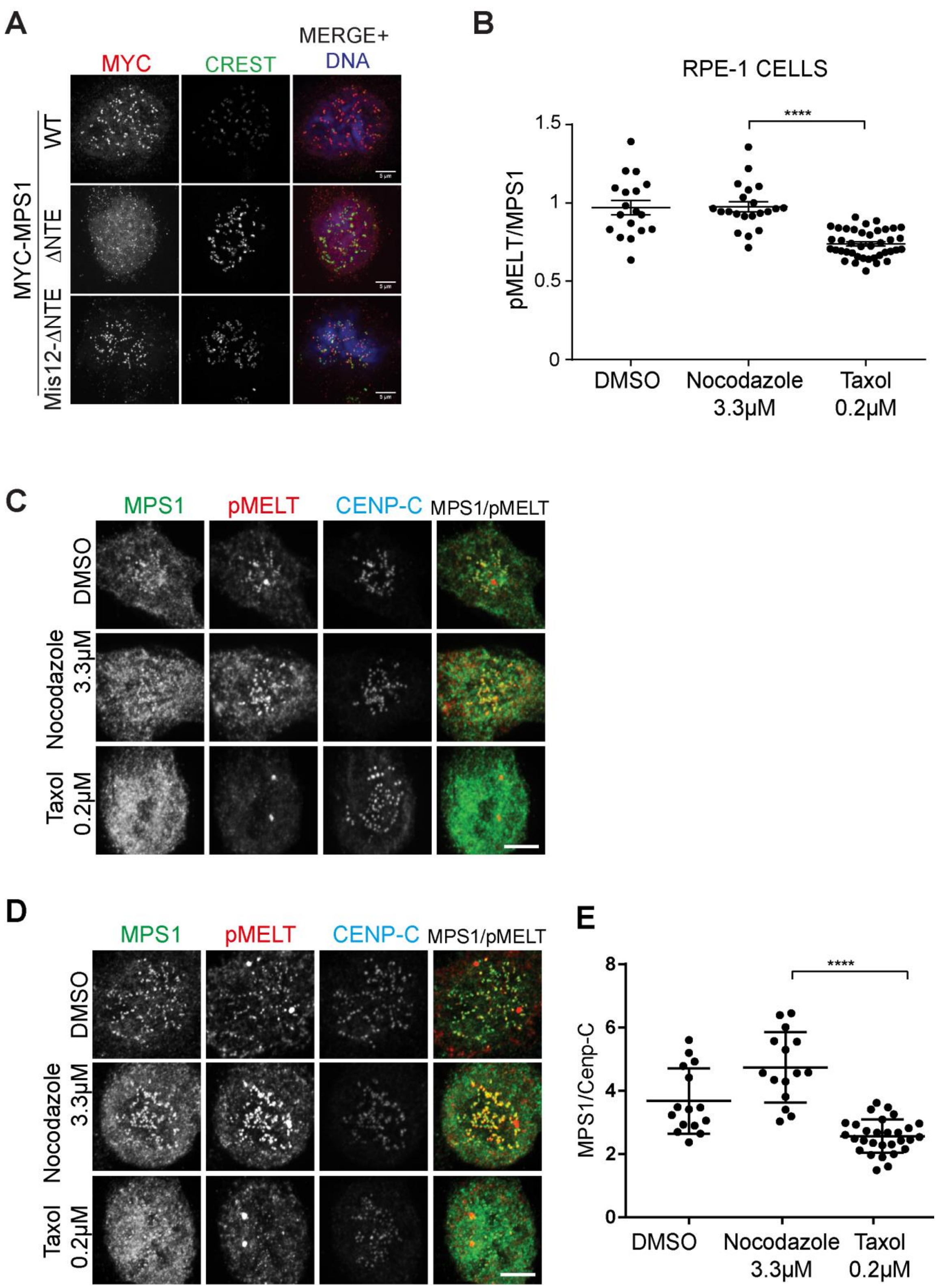

A
MYC
CREST
MERGE+ DNA
MYC-MPS1
WT
ΔNTE
Mis12-ΔNTE
5 µm
B
RPE-1 CELLS
pMELT/MPS1
****
1.5
1
0.5
0
DMSO
Nocodazole 3.3µM
Taxol 0.2µM
C
MPS1
pMELT
CENP-C
MPS1/pMELT
DMSO
Nocodazole 3.3µM
Taxol 0.2µM
D
MPS1
pMELT
CENP-C
MPS1/pMELT
DMSO
Nocodazole 3.3µM
Taxol 0.2µM
E
MPS1/Cenp-C
****
8
6
4
2
0
DMSO
Nocodazole 3.3µM
Taxol 0.2µM

Figure S3: Increased local concentration and transactivation contribute to Mps1 activation, related to Figure 2.

(A) HeLaS3 cells were simultaneously transfected with the indicated constructs and siMps1 and fixed 10h after release from a thymidine arrest. Cells were immunostained against MYC (Red), CREST (Green), and DNA was visualized by Hoechst (Blue). Scale bar = 5µm. **(B)** RPE-1 cells treated for 16h with 3.3µM nocodazole, 0.2µM taxol or DMSO, and then fixed. The graph shows the quantification of the pMELT signal relative to total Mps1. **** $p < 0.0001$. **(C)** Representative images of cells quantified in (B). Cells were immunostained against MYC (Green), pMELT (Red), CENP-C (Cyan) and Hoechst (Blue). Scale bar = 5µm. **(D)** HeLa cells treated for 16h with 3.3µM nocodazole, 0.2µM taxol or DMSO, and then fixed. Cells were immunostained against MYC (Green), pMELT (Red), CENP-C (Cyan) and DNA was marked by Hoechst (Blue). Scale bar = 5µm. See Figure 2F for quantification. **(E)** quantitation of the Mps1 signal versus Cenp-C at kinetochores for the data in Figure 2F.

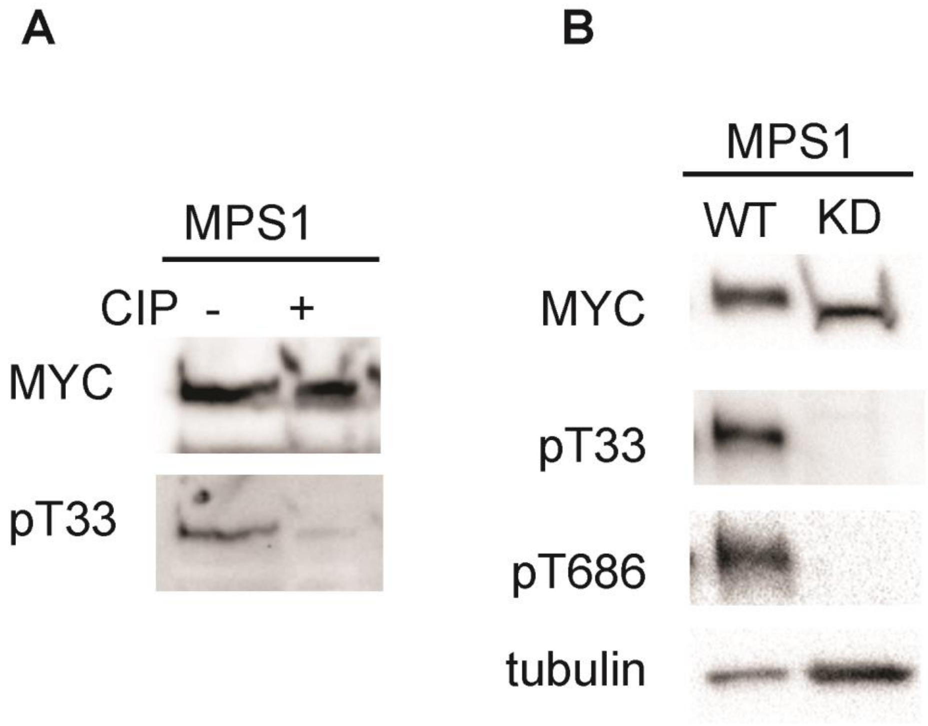

Figure S4. Characterization of the anti-pT33 phosphospecific Mps1 antibody, related to Figure 4.

(A) MYC-Mps1 was immunoprecipitated from nocodazole arrested HEK 293T cells and left untreated or was treated with calf-intestinal phosphatase for 60min at 37°C before the reaction was stopped with SDS-PAGE sample buffer. Resolved samples were probed with pT33 antibodies before stripping and reprobing with MYC. **(B)** Cells expressing MYC-Mps1 -WT and -KD were arrested in mitosis with nocodazole and the MYC-Mps1 proteins were immunoprecipitated from equalized lysates before being resolved by SDS-PAGE and immunoblotted with the indicated antibodies.

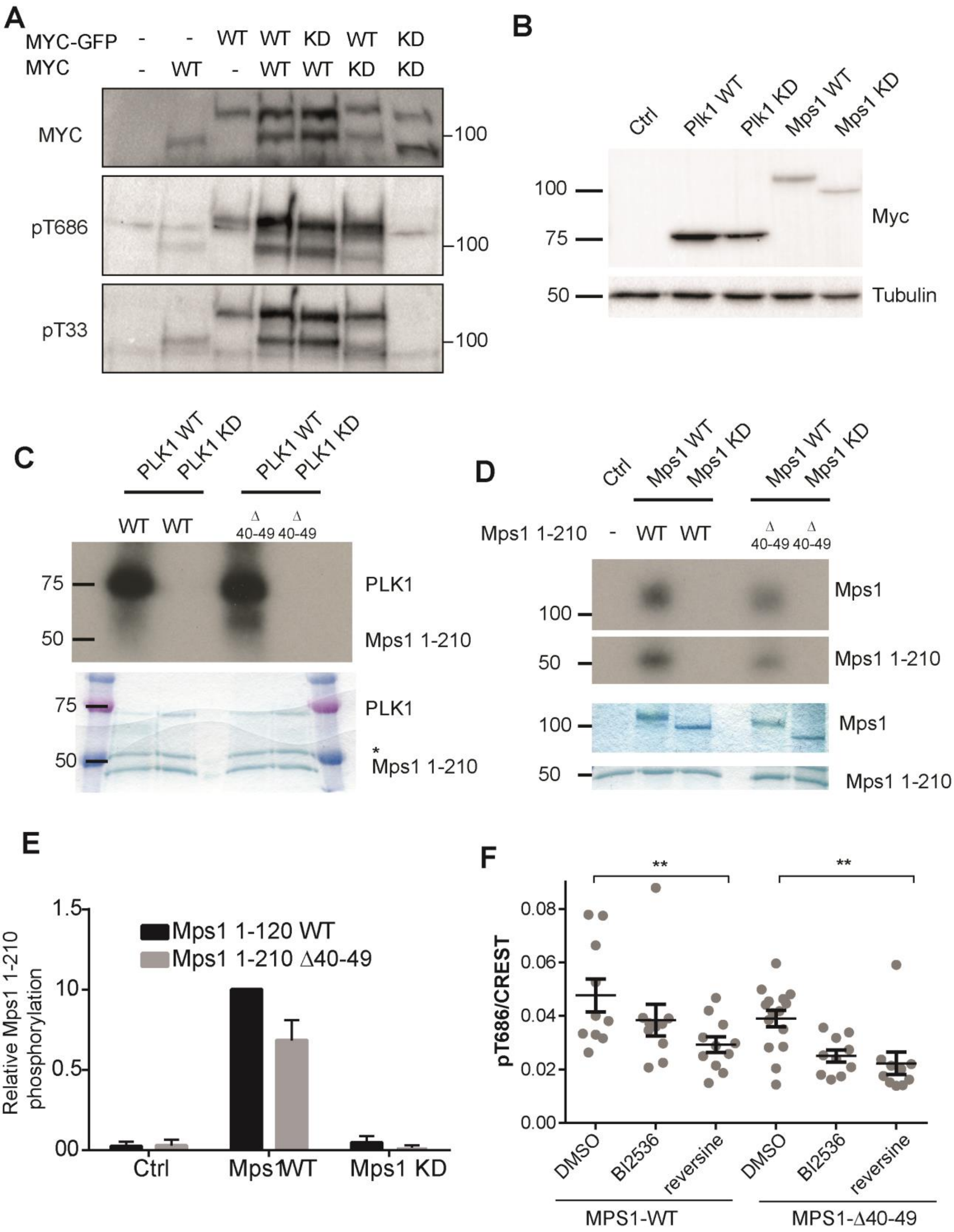

A
MYC-GFP - - WT WT KD WT KD
MYC - WT - WT WT KD KD
MYC
pT686
pT33
100
B
Ctrl
Plk1 WT
Plk1 KD
Mps1 WT
Mps1 KD
Myc
Tubulin
100
75
50
C
PLK1 WT
PLK1 KD
WT
Δ 40-49
PLK1
Mps1 1-210
75
50
D
Ctrl
Mps1 WT
Mps1 KD
Mps1 1-210
WT
Δ 40-49
Mps1
100
50
E
Mps1 1-120 WT
Mps1 1-210 Δ40-49
Relative Mps1 1-210 phosphorylation
Ctrl
Mps1 WT
Mps1 KD
F
pT686/CREST
DMSO
BI2536
reversine
MPS1-WT
MPS1-Δ40-49
**

Figure S5. Relief of Mps1 NTE-mediated autoinhibition does not require significant Plk1 activity, related to Figure 4.

(A) HEK 293T cells were transfected with the indicated Mps1-WT and -KD constructs. Lysates were blotted for T33 and T686 phosphorylation. Reblotting with MYC demonstrates equal loading. **(B)** Expression of Mps1 and Plk1 used in (C) and (D). In vitro kinase assay of Mps1 1-210 and Mps1 1-210 Δ40-49 recombinant fragments using full length Plk1 **(C)** and full length Mps1 **(D)**. **(E)** Quantitation of recombinant Mps1 fragment phosphorylation from (D). **(F)** MYC-GFP-Mps1 WT and Δ40-49 expressing HeLa cells were treated with nocodazole overnight before addition of MG132 and the additional inhibitors as indicated. Fixed cells were stained with MYC, Mps1 pT686, and CREST antisera. Relative quantification of pT686 signals is shown. **$p < 0.01$.

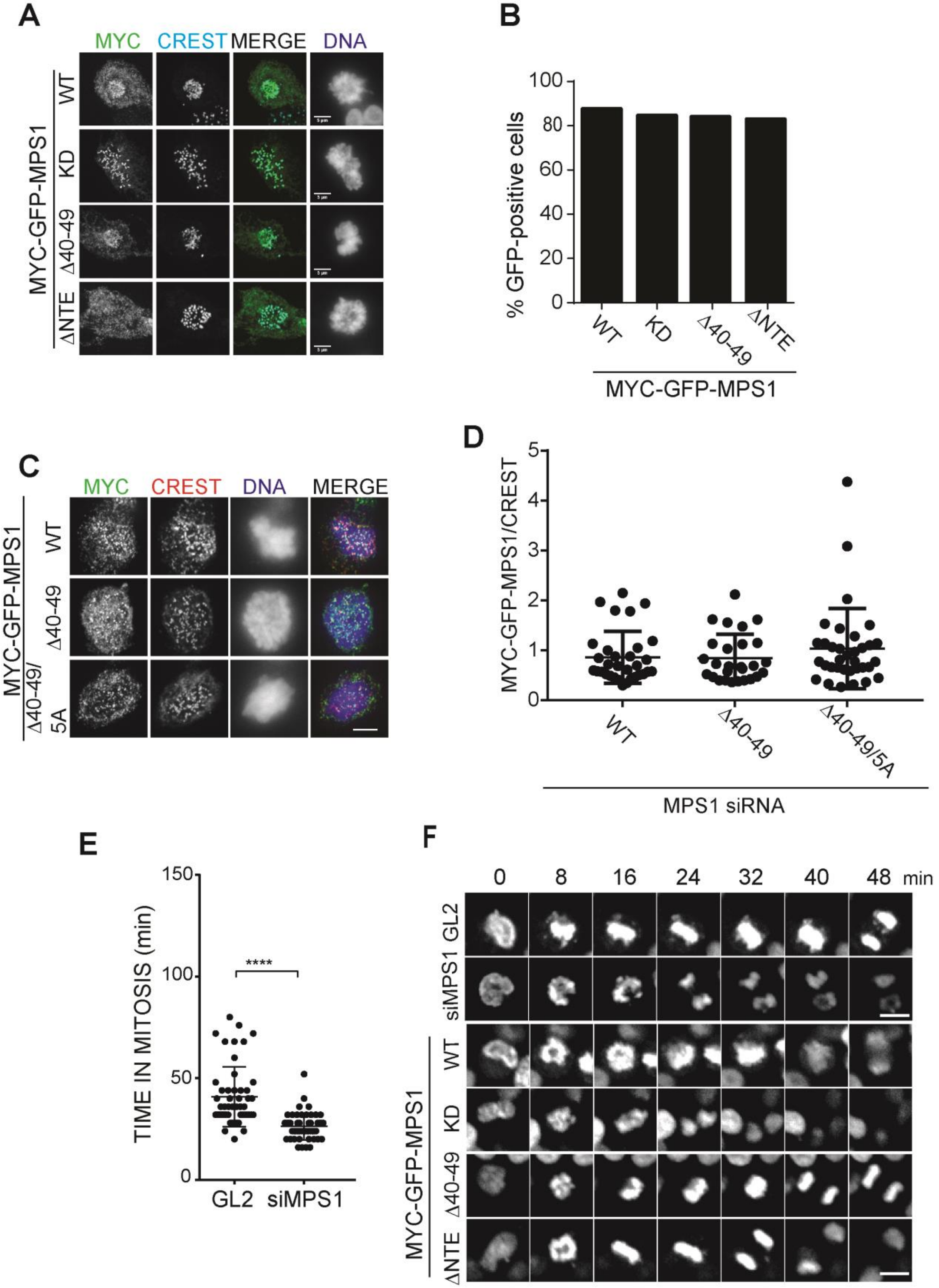

A
MYC
CREST
MERGE
DNA
MYC-GFP-MPS1
WT
KD
Δ40-49
ΔNTE
B
% GFP-positive cells
0
20
40
60
80
100
WT
KD
Δ40-49
ΔNTE
MYC-GFP-MPS1
C
MYC
CREST
DNA
MERGE
MYC-GFP-MPS1
WT
Δ40-49
Δ40-49/5A
D
MYC-GFP-MPS1/CREST
0
1
2
3
4
5
WT
Δ40-49
Δ40-49/5A
MPS1 siRNA
E
TIME IN MITOSIS (min)
0
50
100
150
****
GL2
siMPS1
F
0
8
16
24
32
40
48
min
GL2
siMPS1
MYC-GFP-MPS1
WT
KD
Δ40-49
ΔNTE

Figure S6. Characterization of the HeLa T-Rex stable cell lines used in this study, related to Figure 6.

(A) Immunofluorescence of HeLa T-Rex cell lines expressing 3xMYC-GFP-MPS1 constructs. Cells were stained for MYC (green), CREST (cyan) and DNA was visualized with Hoechst (blue). Scale bar=5µM. **(B)** Graphic representing the percentage of GFP-positive cells of the indicated cell lines. A minimum of 100 cells was counted per cell lines. **(C)** HeLa-T-Rex cell lines stably expressing the indicated constructs were enriched in mitosis by nocodazole treatment before fixation and staining with the indicated antibodies. **(D)** Quantitation of the relative kinetochore intensity of the Mps1 from the HeLa-T-Rex cell lines in (C). **(E)** Time (mins) in mitosis (nuclear envelope breakdown to anaphase onset) of siGL2 and siMPS1 treated cells, the control of the experiment in (Figure 6E). A minimum of 50 expressing cells was counted for each condition. n=2. Scale bar = 10µm. **** $p < 0.0001$. **(F)** Stills from representative cells of each cell line from live-cell experiments in (Figure 6E and Figure S6E).

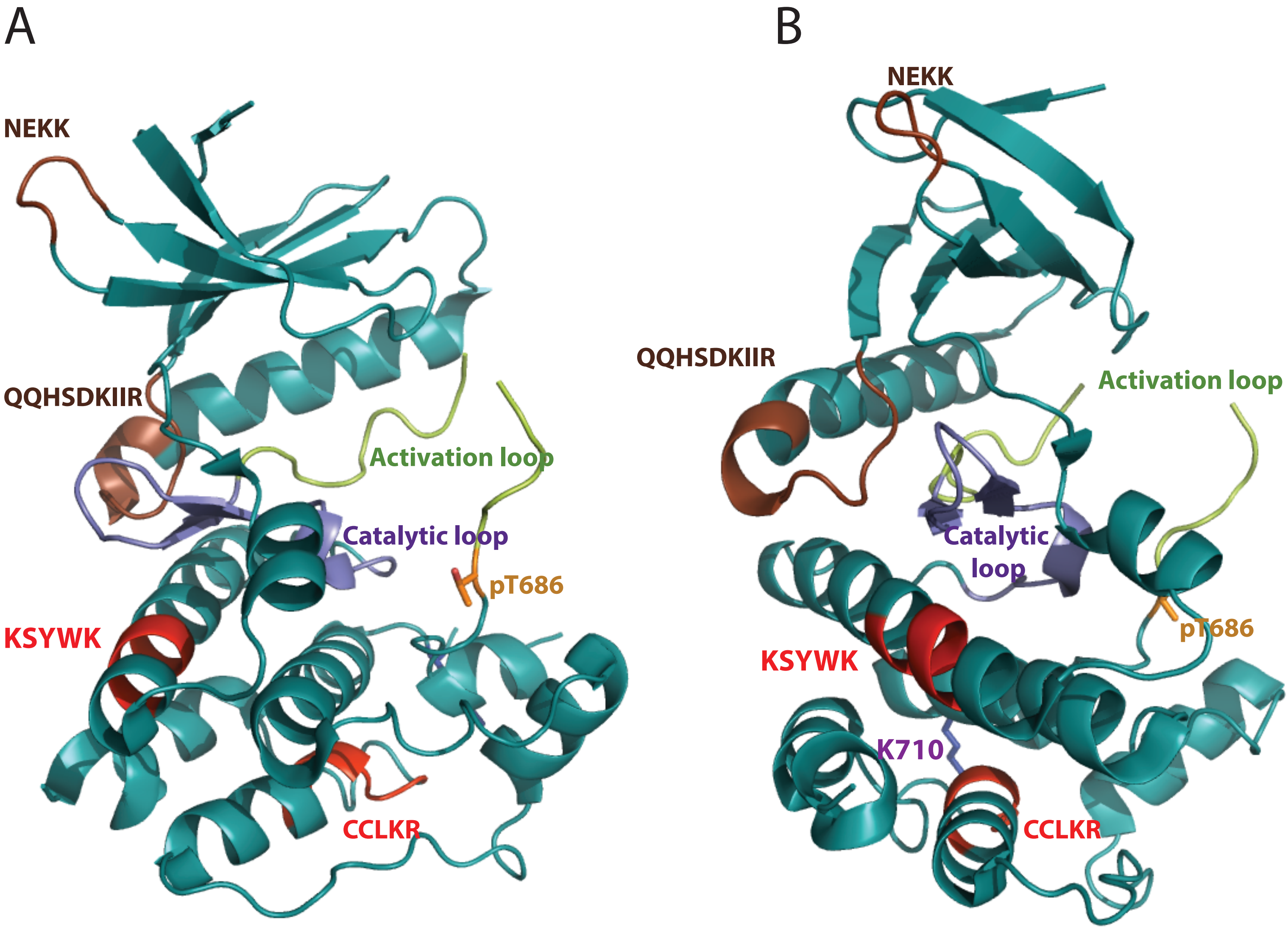
A
NEKK
QQHSDKIIR
Activation loop
Catalytic loop
pT686
KSYWK
CCLKR
B
NEKK
QQHSDKIIR
Activation loop
Catalytic
loop
pT686
KSYWK
K710
CCLKR

Figure S7. Mps1 NTE and TPR crosslinks mapped onto the Mps1 kinase domain, related to Figure 7.

Unique crosslinks identified between the NTE and the kinase domain of Mps1 (brown) and the TPR and the kinase domain of Mps1 (red) are mapped on to the structure of the inhibited Mps1 kinase domain (PDB ID: 2ZMC). The catalytic loop is shown in blue whereas the activation loop is in green. **(A)** and **(B)** are rotated by 45º. Mapping the kinase domain residues identified in the cross-links with the NTE (and TPR) onto the Mps1 kinase structure suggests that the NTE region interacts with the N-terminal lobe region that is defined by the residues V^{539}FQVLNEKK^{547} and Q^{579}QHSDKIIR^{587} (numbering according to Mps1 isoform 1, NCBI NP_003309.2). The crystal structure of Mps1 kinase domain in its inhibited conformation shows that residues of the Q^{579}QHSDKIIR^{587} region form part of α-helix C and are in close spatial proximity (eg, 5 Å or less) to the activation and the catalytic loops. The C-terminal lobe region that is defined by the residues K^{625}SYWK^{629} and the more C-terminal region C^{770}CLKR^{774} both interact with the TPR motif. The activation loop (residues 672-680) and the loop between helices αF and αEF (residues 700-708) are flexible and disordered. The residues K^{625}SYWK^{629} form part of αE helix and are in close proximity to the catalytic loop (particularly the side chain of Tyrosine residue Y627). Because the C^{770}CLKR^{774} residues form part of αH helix, the intramolecular interaction of the TPR motif with the kinase domain may affect the local conformation of the activation loop, thus contributing to modulate Mps1 catalytic activity. Two positively charged residues, K708 and K710, which are mapped onto the loop connecting αF and αEF and in close proximity to the P+1 and the activation loops, stabilize the active conformation.
